# Unlocking the Potential of Mg-Doped Rare Earth Manganites: Machine Learning-Guided Synthesis and Insights into Structural and Optical Properties

**DOI:** 10.3390/nano15070561

**Published:** 2025-04-06

**Authors:** Chikh Lakhdar Ben Messaoud, Zoulikha Hebboul, Ibn Khaldoun Lefkaier, Ahmed Draoui, Ahmed Lamine Ben Kamri, Souraya Goumri-Said, Mohammed Benali Kanoun, Romualdo S. Silva, José A. Alonso, Sophie Laurent

**Affiliations:** 1 Laboratoire de Physique des Matériaux, Ammar Thelidji, University of Laghouat, Laghouat 03000, Algeria; c.benmessaoud@lagh-univ.dz (C.L.B.M.); ik.lefkaier@lagh-univ.dz (I.K.L.); a.draoui@lagh-univ.dz (A.D.);; 2 Laboratoire de Physico-Chimie des Matériaux, Ammar Thelidji University of Laghouat, Laghouat 03000, Algeria; z.hebboul@lagh-univ.dz; 3 Department of Physics, College of Science and General Studies, Alfaisal University, P.O. Box 5092, Riyadh 11533, Saudi Arabia; 4 Department of Mathematics and Sciences, College of Humanities and Sciences, Prince Sultan University, P.O. Box 66833, Riyadh 11586, Saudi Arabia; mkanoun@psu.edu.sa; 5 Instituto de Ciencia de Materiales de Madrid (ICMM), CSIC, E-28049 Madrid, Spain; romualdo.silva@csic.es (R.S.S.J.); ja.alonso@icmm.csic.es (J.A.A.); 6 Department of General, Organic, and Biomedical Chemistry, NMR and Molecular Imaging Laboratory, University of Mons-Hainaut, B-7000 Mons, Belgium; sophie.laurent@umons.ac.be

**Keywords:** nanomanganites, perovskite, Mg doping, sol–gel, SEM, gap energy, multifunctional materials

## Abstract

By leveraging machine learning insights from prior perovskite studies and employing the sol–gel method, we successfully synthesized two novel perovskite nanoceramics—M_0.5_ Ca_0.25_Mg_0.25_MnO_3_ (M = La, Pr)—as multifunctional nanomaterials. X-ray diffraction (XRD) confirmed their orthorhombic Pnma crystal structure. The Williamson–Hall method estimated average particle sizes of 59.5 nm for PCMMO and 21.8 nm for LCMMO, while the Scherrer method provided corresponding values of 32.59 nm and 20.43 nm. SEM, UV-Vis, and FTIR analyses validated the chemical composition, homogeneity, and optical properties of the synthesized compounds, revealing band gaps of 3.25 eV (LCMMO) and 3.71 eV (PCMMO) with Urbach energies of 0.29 eV and 0.26 eV, respectively. These findings provide valuable insights into the structural and optical properties of LCMMO and PCMMO, highlighting their potential as multifunctional materials for advanced device applications.

## 1. Introduction

The development of materials is a fundamental pillar of scientific and technological progress. With the advancement in machine learning (ML) techniques, these tools have become an effective means for analyzing and designing materials in innovative ways. Machine learning is distinguished by its ability to extract relationships from data without relying on traditional physical mechanisms, enabling the prediction of virtual material properties and their evaluation to discover high-performance materials. In light of our research team’s recent focus on machine learning [1,2,3], particularly the latest work by Soundous [4] and two other related studies in this field [5,6], inspiring ideas have emerged regarding ABX_3_ perovskite materials. The results of these studies drew our attention to the fascinating properties of manganite perovskite compounds, including magnetic properties, insulating properties, and other intriguing characteristics, making them a central focus for scientific research and technological applications.

Perovskite manganites with the general formula R_1−x_A_x_MnO_3_ (where R is a trivalent rare-earth ion and A is a divalent alkaline-earth) have attracted the attention of researchers and scientists due to the intricate interplay between spin, charge, orbital, and lattice degrees of freedom, which gives rise to their remarkable properties [7,8]. These materials exhibit a wide range of phenomena, including colossal magnetoresistance (CMR) [9,10], metal–insulator transitions, charge/orbital ordering [11,12], and various magnetic phases such as paramagnetism, super-paramagnetism, ferromagnetism, antiferromagnetism, and spin-glass behavior. The parent compounds, RMnO_3_, are antiferromagnetic insulators [13,14], but doping with divalent cations like Mg^2+^, Ca^2+^, Sr^2+^, or Ba^2+^ at the R-site induces mixed Mn^3+^/Mn^4+^ valence states, which dramatically affects their magnetic and related transport properties. The double-exchange mechanism, initially proposed by Zener [11,15] and later refined by others [16,17], provides insight into the correlation between ferromagnetic ordering and conductivity observed in these systems. According to this mechanism, the mobility of electrons is maximized when neighboring Mn^3+^/Mn^4+^ spins align in parallel, promoting ferromagnetic ordering and metallic behavior. The exceptional properties of perovskite manganites make them promising for various technological applications, including random-access memories [18,19], colossal magnetoresistance (CMR) [9,20], the magnetocaloric effect (MCE) [21,22], photocatalysis [23], magnetic refrigeration [24], electric field sensors [25], spintronics [26], and photovoltaic materials [27]. Additionally, some compositions show multiferroic behavior [28,29], combining ferroelectric and ferromagnetic order, which enables multifunctional device applications. Extensive research on La_1−x_Ca_x_MnO_3_ (LCMO) systems has revealed complex phase diagrams characterized by diverse magnetic and electronic phases. Phases such as ferromagnetic metallic (FM-M), charge-ordered (CO) antiferromagnetic insulating, and canted antiferromagnetic (CAF) emerge based on Ca doping levels and temperature. Strong electron correlations in these systems complicate a detailed microscopic understanding of their properties. Notably, a significant phase transition occurs from a low-temperature FM-M phase to a high-temperature paramagnetic insulating (PM-I) phase for doping levels between 0.2 < x < 0.5 [30]. 

In recent years, considerable attention has focused on the effects of finite size and surface modifications in nanoscale perovskite manganites [31,32]. Studies have shown that reducing particle size can significantly impact magnetism and transport characteristics by destabilizing charge and orbital order, leading to notable differences from bulk counterparts. This phenomenon, especially relevant at the nanoscale, has sparked substantial interest. Two primary hypotheses aim to explain these size-dependent effects: modifications to bulk strain and changes in surface electronic structure [33,34,35,36]. Further studies are needed to clarify the competing factors influencing nanoscale materials. Perovskite manganite nanoparticles exhibit unique properties, including super-paramagnetism with potential applications in magnetic hyperthermia [37,38,39], surface spin-glass behavior, large coercivities, reduced Curie temperatures, and low-field saturation magnetization compared to their bulk counterparts [40,41]. Ongoing research explores the effects of different synthesis methods, doping strategies, and nanostructuring on the physical properties of these manganites [42,43,44,45].

Although extensive research has focused on the magnetic and transport properties of manganites, their optical properties have received less attention, primarily due to their insulating (large bandgap) or metallic (no bandgap) behavior [46]. However, if these systems could be induced to exhibit semiconducting characteristics along with strong magnetic moments, they could become promising candidates for magnetic semiconducting devices.

While there is substantial research on LCMO (La_1−x_Sr_x_MnO_3_) and LSMO (La_1-x_Sr_x_MnO_3_) systems, other compositions within the R_1−x_A_x_MnO_3_ family, such as Pr_1−x_Ca_x_MnO_3_ (PCMO) [47,48,49] and Pr_1−x_Sr_x_MnO_3_ (PSMO) [50,51], have received comparatively less attention. These systems have distinct phase diagrams and physical properties that warrant further investigation, particularly in the high doping regime (x > 0.5) where charge ordering and canted antiferromagnetism are expected to play significant roles.

Although numerous studies on the doping of LaMnO_3_ with magnesium have been conducted, firstly by X. Z. Zhou [52] and Li [53] and recently by Selmi [54]. However, no similar investigations have been made to explore the effects of magnesium on the La_1−x_Ca_x_MnO_3_ and Pr_1−x_Ca_x_MnO_3_ compounds. The existing literature in this area is limited to a single study by L. Liu et al. [55], who employed oxides and carbonates (La_2_O_3_, CaCO_3_, MgO, and MnCO_3_) as reactants and used the chemical composition La_2_/_3_(Ca_1−x_Mg_x_)_1_/_3_MnO_3_ (with x ranging from 0 to 0.5). In contrast, Garcia et al. [56] demonstrated that doping LaMnO_3_ with magnesium could enhance its semiconducting properties, with the structure of all the samples belonging to space group R-3c. Similar results are observed in other systems such as ZnO [57,58]. For this purpose, studying the substitution of magnesium in La_1−x_Ca_x_MnO_3_ and Pr_1−x_Ca_x_MnO_3_ is essential for evaluating its potential to induce significant changes in the structural and functional properties of LCMMO and PCMMO materials, they possess significant potential to serve as exceptional materials for advanced magnetic semiconductor technologies.

Based on the findings from machine learning studies related to perovskites and the unique properties of the La_1−x_Ca_x_MnO_3_ compound, such as magnetism, colossal magnetoresistance (CMR), and their importance in spintronic and insulating applications, along with Garcia’s study that indicated magnesium doping could enhance semiconductor properties, and our semi-comprehensive research (see Table S1), we select to prepare and characterize two new nanomaterials: La_0.5_Ca_0.25_Mg_0.25_MnO_3_ (LCMMO) and Pr_0.5_Ca_0.25_Mg_0.25_MnO_3_ (PCMMO). The innovation lies in substituting magnesium into the chemical composition to explore its effect on their physical properties. The study also included a systematic comparison of the impact of lanthanum (La) and praseodymium (Pr) substitution to gain a deeper understanding of these modification effects. This study aims to leverage machine learning results along with experimental observations to prepare innovative materials that enhance semiconductor properties while maintaining the well-known magnetic properties, contributing to a broader understanding of these materials’ behavior and opening new avenues for advanced technological applications.

## 2. Exploring Materials: Advanced Research Techniques

The reactants were sourced from Sigma–Aldrich (St. Louis, MO, USA)with high purity, including Manganese (II) nitrate tetrahydrate (Mn(NO_3_)_2_·4H_2_O 99.9%), lanthanum nitrate hexahydrate (La(NO_3_)_3_·6H_2_O 99.99%), praseodymium (III) nitrate hexahydrate (Pr(NO_3_)_3_·6H_2_O 99.9%), calcium nitrate tetrahydrate (Ca(NO_3_)_2_·4H_2_O 99%), and magnesium nitrate anhydrous (Mg(NO_3_)_2_ 99%). Powder X-ray diffraction (XRD) patterns of the final products were obtained using a Panalytical EMPYREAN (Malvern, UK) powder diffractometer (CuK 1, 40 mA, 30 kV) with a step size of 0.01° and an acquisition time of 6 s/step in the 15–70° 2θ range under room temperature conditions. The nanoparticle size distribution and chemical composition were examined using scanning electron microscopy (SEM) with a TESCAN VEGA3 SBU EasyProbe electron microscope system (Brno, Czech Republic) coupled with a Bruker detector (Billerica, MA, USA) for energy-dispersive X-ray (EDX) analysis. The molar concentrations of lanthanum, praseodymium, calcium, and magnesium were determined using the ESPRIT Microanalysis Software (v2.5) from Bruker. Secondary electron images were acquired using primary electron beams at energies of 5 keV and 25 keV. The elemental samples were analyzed using an X-ray fluorescence (XRF) spectrometer (S2PUMA-BRUKER, Karlsruhe, Germany) with energy-dispersive X-ray fluorescence (EDXRF) capabilities. This spectrometer employs a Pd source for generating X-rays and can automatically adjust its focus points between 0.3 and 10 mm in diameter using a silicon drift detector. The sample chamber can operate in air, helium (He), nitrogen (N2), or vacuum environments. The voltage applied can go up to 40 kV. The optical properties, the band gap and Urbach energies of the samples, were explored by ultraviolet-visible spectroscopic analysis (UV-VIS) using a Shimadzu UV1800 (Kyoto, Japan) spectrophotometer. The FTIR measurements were conducted using a Jasco FT/IR-4200 instrument (Easton, MD, USA), the FTIR spectrum was recorded in transmission mode over the range of 2500–400 cm^−1^, utilizing a KBr pellet as the sample matrix.

## 3. Strategic Insights into Prepared Samples Selection

Recent machine learning studies on perovskite materials [4,5] have identified manganese perovskite compounds as among the most promising due to their diverse applications and technological potential. Given the increasing interest in these materials, we conducted research to select and characterize new nanomaterials, focusing on addressing research gaps and enhancing their fundamental properties for broader applications. Substitutions at the A-site in these compounds play a crucial role in determining their structural, chemical, and physical properties. By incorporating alkaline-earth and rare-earth elements, the average ionic radius of the A-site can be modified, leading to structural changes that influence their optical and dielectric properties [46,55,59]. As shown in Table S1, this table serves as a key reference for the samples prepared and characterized based on prior research. It presents three scenarios for synthesizing new predicted materials, focusing on variations in the A-site element position. The first scenario, shown in column 1, involves combining one rare-earth element with one alkaline-earth element. Columns 2 and 3 outline two additional scenarios: the second uses two alkaline-earth elements with one rare-earth element, while the third uses two rare-earth elements with one alkaline-earth element. In Table S1, the green highlights indicate materials that have not been synthesized or characterized yet, representing 66.67% (40 out of 60) of the total. The orange highlights correspond to materials with limited research, accounting for 13.33% (8 out of 60), while red signifies materials that have been extensively studied, making up 20% (12 out of 60) of the materials.

Leveraging the ionic radii of selected elements from Table S2, derived from Shannon’s ionic radius table, and utilizing Goldschmidt’s equation (Equation (S1)), alongside Equations (S2) and (S3) for the calculation of the average ionic radius of the A-site cations, we determined the tolerance factors for 60 perovskite manganite solid solution compounds. These tolerance factors enabled the prediction of the crystal structures for each compound, as presented in Table S3. The tolerance factor serves as a pivotal parameter for assessing the stability of perovskite crystal structures, with values approaching unity denoting enhanced structural stability. The computed tolerance factors for the compounds span from 0.85 to 0.98, highlighting diverse structural preferences. Specifically, compounds with tolerance factors between 0.95 and 0.99 are predisposed to adopting rhombohedral phases, whereas those with factors below 0.95 exhibit a tendency toward orthorhombic phases. Noteworthy is the incorporation of Barium (Ba), whose larger ionic radius relative to calcium (Ca) and magnesium (Mg) induces rhombohedral distortion, thereby significantly influencing the crystal structure and broadening the applicability of these materials in various technological contexts. The analysis further indicates that orthorhombic structures are the predominant predicted phase, comprising 70% of the materials, while rhombohedral phases account for the remaining 30%, as depicted in Figure S1.

In light of this, we focused on the synthesis and characterization of two novel nanomaterials: La_0.5_Ca_0.25_Mg_0.25_MnO_3_ (LCMMO) and Pr_0.5_Ca_0.25_Mg_0.25_MnO_3_ (PCMMO). Our investigation prioritized their optical properties to evaluate their potential for semiconductor applications.

## 4. Results and Discussion

### 4.1. Preparation

**Figure 1:**
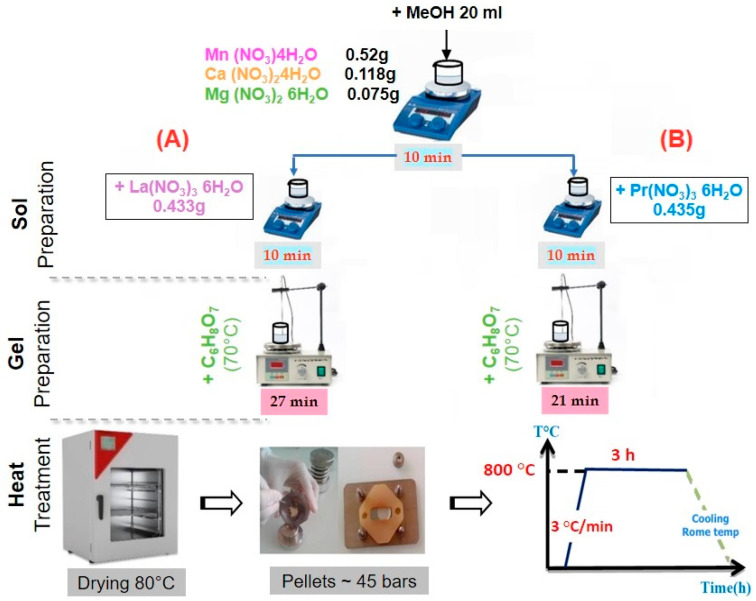
Sample experimental protocol: (**A**) LCMMO; (**B**) PCMMO.

New nanocrystalline samples with targeted compositions of La_0.5_Ca_0.25_Mg_0.25_MnO_3_ (LCMMO) and Pr_0.5_Ca_0.25_Mg_0.25_MnO_3_ (PCMMO) were prepared using the sol–gel technique. In the first step, the reagents including Mn(NO_3_)_2_·4H_2_O (2.07 mmole), Ca(NO_3_)_2_·4H_2_O (0.5 mmole), and Mg(NO_3_)_2_ (0.5 mmole) were dissolved in methanol (MeOH) solvent using a magnetic stirrer for 10 min. Then, La(NO_3_)_3_·6H_2_O (1 mmole) and Pr(NO_3_)_3_·6H_2_O (1 mmole) were added separately using a magnetic stirrer for 10 min for the second time in sol preparation (A) and (B), respectively. A citric acid (C_6_H_8_O_7_) solution (0.6 g in MeOH) was added to the mixture. The key roles of citric acid in the sol–gel process are (i) as a chelating agent for metal ions, (ii) as a viscosity-controlling agent of the medium, and (iii) as an organic fuel during the calcination process. In the second step, the resulting solutions were heated to 70 °C until gel formation occurred in 27 min (LCMMO preparation) and 21 min (PCMMO preparation), respectively. Finally, the gels were dried at 80 °C in a drying cabinet for 24 h to produce precursor powders. The obtained powders were milled and annealed at 800 °C for 2.5 h under ambient air conditions (Figure 1).

### 4.2. Morphology and Elemental Composition

**Figure 2:**
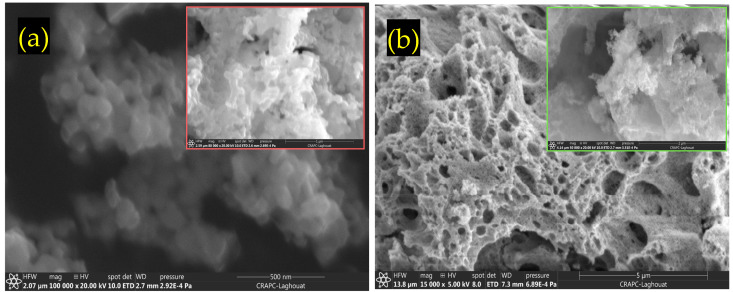
SEM images for (**a**) LCMMO and (**b**) PCMMO nano powders.

**Figure 3:**
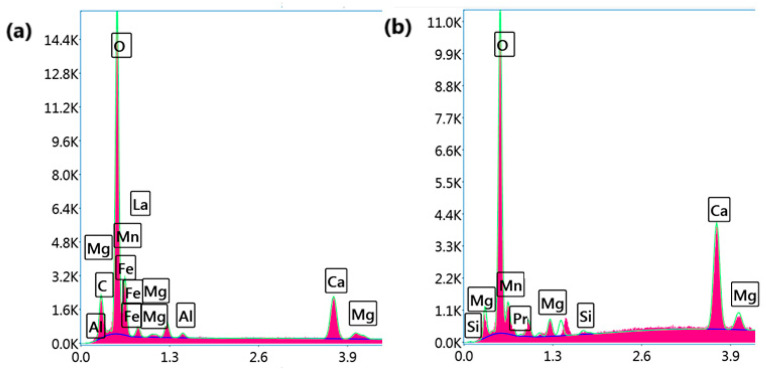
EDX mappings and spectra for (**a**) LCMMO and (**b**) PCMMO nanoparticles.

Figure 2 presents SEM micrographs of (a) LCMMO and (b) PCMMO. Although the resolution limitations of the SEM prevented detailed imaging at the nanoscale, it effectively revealed the aggregated morphology of the nanoparticles. Both samples exhibit a uniform polycrystalline and porous structure composed of densely packed grains—a typical microstructural trait of perovskites synthesized via the sol–gel method followed by thermal treatment. The powders were successfully characterized in terms of chemical composition and homogeneity. The SEM images reveal micron-sized spherical agglomerates composed of finer nanoparticles, with some aggregates measuring below 100 nm, as highlighted in the insets of Figure 2a,b. Energy-dispersive X-ray spectroscopy (EDX) analysis confirmed the elemental composition, phase purity, and stoichiometry of the samples, closely aligning with the intended molar ratio of 0.5:0.25:0.25:1 for La (or Pr), Ca, Mg, and Mn, respectively. These results were averaged over multiple zones to ensure consistency and representativeness. No notable impurities were observed in either sample. The apparent Fe peak observed in Figure 3a is attributed to the software limitations of the EDX detector, where closely overlapping signals from Mn and Fe can lead to erroneous peak identification. Minor unidentified signals are attributed to the sample substrate. Furthermore, the X-ray fluorescence (XRF) analysis corroborated the expected stoichiometry and elemental distribution, as summarized in Table 1.

### 4.3. XRD Characterization

A. L. Liu et al. [55] studied a variant composition of (La_2_/_3_(Ca_1−x_Mg_x_)_1_/_3_MnO_3_) emphasizing the enhanced durability of the orthorhombic perovskite lattice when subjected to compositional alterations. The parent compounds of our study are La_0.5_Ca_0.5_MnO_3_ [60] and Pr_0.5_Ca_0.5_MnO_3_ [61,62], both of which are isostructural and characterized by the same crystal structure. Their powder diffraction profiles are identified in the ICDD Cards No. 01-089-0793 and No. 01-089-0795, respectively.

**Figure 4:**
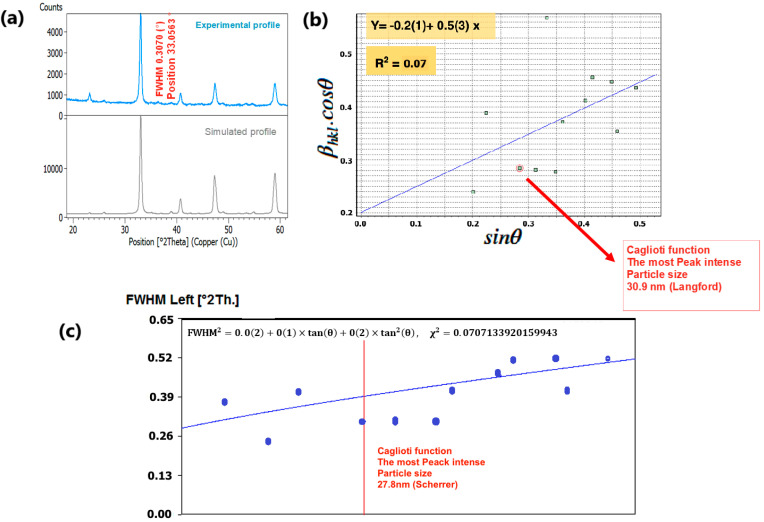
(**a**) XRD spectrum of LCMMO, (**b**) Williamson–Hall plot, and (**c**) FWHM Caglioti function.

**Figure 5:**
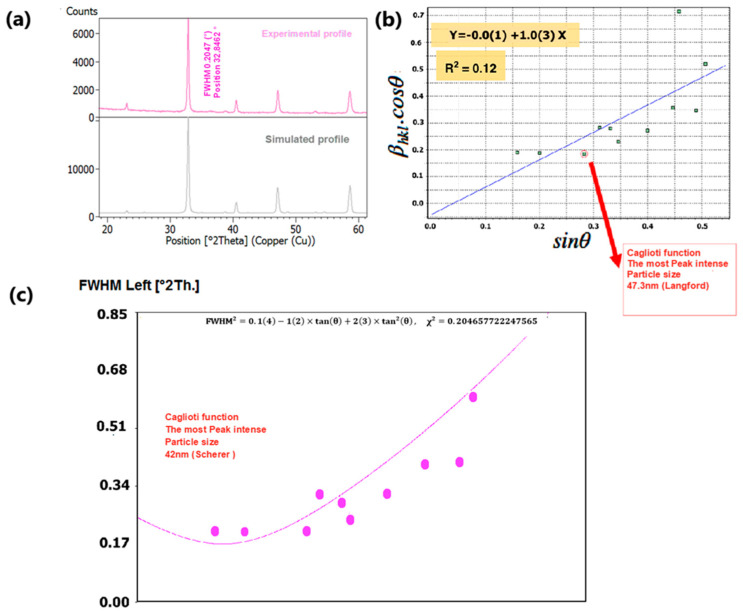
(**a**) XRD spectrum of PCMMO, (**b**) Williamson–Hall plot, and (**c**) FWHM Caglioti function.

To delve into the nuances of structural variations induced by changes in composition, we meticulously examined the phase purity and crystal structure of our nano manganite powders, which boast distinct particle sizes. This scrutiny was accomplished through an X-ray diffraction (XRD) analysis conducted at room temperature. The XRD findings, as depicted in Figure 4a and Figure 5a, underscored the presence of a pristine orthorhombic perovskite crystalline structure in both LCMMO and PCMMO, corroborating their conformity to the Pnma space group (experimental profile). Despite the doping of Mg, there was no discernible alteration in the appearance or disappearance of diffraction peaks (simulated profile). The powder XRD patterns of the investigated manganite perovskites LCMMO and PCMMO reveal well-crystallized nanoparticle phases and showcase particle size uniformity through Williamson–Hall plots in Figure 4b and Figure 5b. The shapes of peaks were modeled using a pseudo-Voigt function and a Caglioti model [63] and Scherrer’s formula using X’Pert HighScore Plus. The XRD patterns were originally generated using the HighScore software (version 5.0). For the Williamson–Hall (W–H) analysis and Caglioti curve fitting, 12 diffraction peaks within the 20–60° 2θ range were selected for LCMMO (Figure 4c), and 11 peaks for PCMMO (Figure 5c).

Regarding the dependence of crystallite size on the annealing process, it is important to note that the final annealing temperature plays a significant role in grain growth. Higher annealing temperatures typically enhance atomic diffusion, which in turn leads to an increase in crystallite size.

The Caglioti equation is given by FWHM^2^ = U·tan^2^θ + V·tanθ + W, where U, V, and W are empirical fitting parameters, and θ is the Bragg angle.

The difference in the linearity of the plots in Figure 4c (linear) and Figure 5c (non-linear) stems from variations in these parameters. These discrepancies reflect underlying microstructural differences and strain variations between the two materials. Figure 4c and Figure 5c). No significant crystallized impurities are detected, as demonstrated by the homogeneity of particle sizes in the Williamson–Hall plots for both PCMMO and LCMMO nanoparticles. In this work, the average particle size of PCMMO was determined to be 59.5 ± 3 nm using the Williamson–Hall (W–H) size–strain analysis (Langford method), with a corresponding strain of 0.2%. This strain value, which falls within the typical range of 0.1–0.5%, suggests the presence of lattice distortions and intrinsic defects. The crystallite size estimated by the Scherrer formula was 32.59 ± 2 nm, which is significantly smaller than that obtained by the W–H method. Since the Scherrer method often underestimates particle size due to its neglect of strain effects, we propose that the W–H result is closer to the true particle size. For LCMMO, the average particle size estimated using the W–H method was 21.8 ± 2 nm, while the Scherrer method yielded a crystallite size of 20.43 ± 1.5 nm. The strain calculated in this case was only 0.02%, indicating high crystallinity and low defect density. This behavior is consistent with the findings in oxide nanoparticles such as TiO_2_ and CeO_2_, where it has been shown that synthesis methods significantly influence structural properties, including crystallinity and strain [64]. These results suggest that the sol–gel synthesis route is particularly effective for preparing highly crystalline LCMMO nanoparticles. The average particle sizes are 59.5 ± 3 nm for PCMMO and 21.8 ± 2 nm for LCMMO compared to crystallite sizes of 32.59 ± 2 nm and 20.43 ± 1.5 nm, respectively, as summarized in Table 2.

### 4.4. Exploring Optical Characteristics

#### 4.4.1. Fourier Transform Infrared Spectroscopy

**Figure 6:**
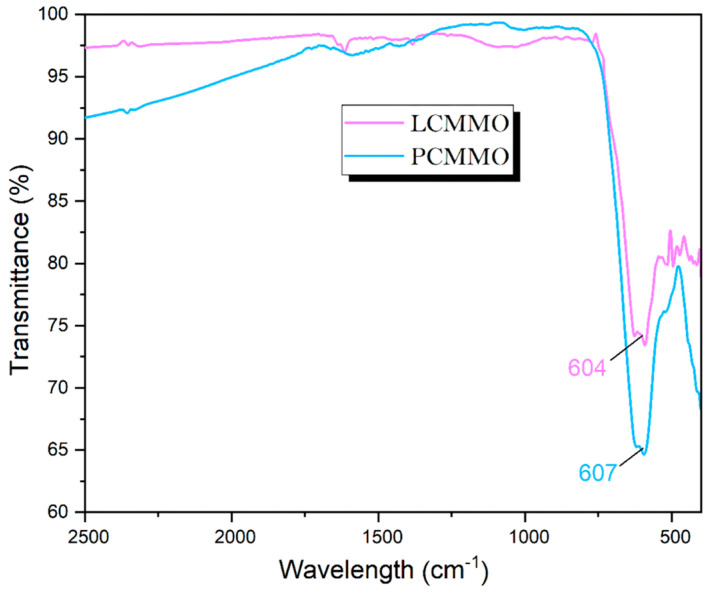
Standard FTIR spectra analysis of LCMMO and PCMMO.

Also known as vibrational spectroscopy (IR), involves a meticulous examination of how infrared light interacts with molecules. In the literature, FIR spectra have been documented for various compounds, including ABX_3_ perovskite titanates [65], niobates [66], fluorides [67], zirconates [68], chromites, ferrites [69], and silicates [70], consistently revealing three absorption bands (around 200 cm^−1^, 400 cm^−1^, and 600 cm^−1^), which are called “external,” “bending,” or “stretching” modes [71], irrespective of stoichiometry and structural variations. Different studies have described the order of these bands in varying ways. The crystal structure of rare-earth manganites typically adopts a distorted GdFeO_3_-type structure, featuring a central Mn atom surrounded by six oxygen ions arranged in an octahedral configuration. While the MnO_6_ octahedron theoretically harbors six vibrating modes, only two exhibit infrared activity. The FTIR spectra depicted in Figure 6 enable the identification of frequency band peaks for both the LCMMO and PCMMO nano powders. Notably, a distinct band around 604 cm^−1^ for LCMMO and 607 cm^−1^ for PCMMO is attributed to the stretching vibration of the O–Mn–O bond within the MnO_6_ octahedron [68]. This indicates that each sample contains a significant presence of the Mn–O bond, and variations in the Mn–O–Mn bond length, driven by internal motion, are responsible for the formation of this band. The confirmation of the O–Mn–O bond presence around 600 cm^−1^ serves to validate the formation of nanocrystallites, consistent with previous research findings. The prominent broad peak observed in nano samples aids in discerning localized distortion within the MnO_6_ octahedral structure in perovskite samples, a critical determinant of their transport. Throughout the synthesis of nano perovskites, various physical effects, including atomic disorder, contamination, and grain boundary dislocations, may significantly influence the structural characteristics and material properties.

#### 4.4.2. UV–Vis Absorbance Spectroscopy

**Figure 7:**
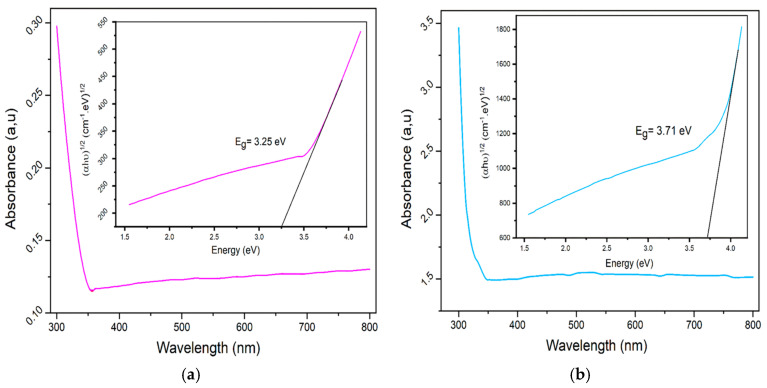
Uv-v absorbance spectra (insert gap energy with Tauc plot) of (**a**) LCMMO and (**b**) PCMMO.

To thoroughly investigate the optical properties of the samples, UV-Vis spectroscopy was employed, a widely used analytical technique that aids in determining absorbance and energy gaps in synthesized nanoparticles. Figure 7 illustrates the UV-Vis absorption intensity of the samples within the 300–800 nm wavelength range. Due to the high concentration of the solid solution, the absorption intensity is notably high. The peak absorption for the LCMMO and PCMMO nanoparticles occurs around 300 nm, indicating that both pure nanoceramics absorb in the UV range. This absorption peak is likely due to a *p-d* charge transfer transition [O(2p)→Mn(3d)] within the MnO_6_ octahedral centers in both perovskites. The optical bandgaps of the LCMMO and PCMMO nanoparticles were derived using Tauc’s equation:

$$\alpha h\nu =A{\left(h\nu -E_{g}\right)}^{n}$$

where

α is the absorption coefficient;

ν represents the energy of the incident light;

A is the absorption edge width;

$E_{g}\;$is the optical bandgap of the material;

n is an index related to different electronic transitions.

By extrapolating the linear portion of the Tauc plot (Figure 7, inset), the optical bandgaps for the LCMMO and PCMMO nanoparticles were estimated at 3.25 ± 0.04 eV and 3.71 ± 0.07 eV, respectively. These values are significantly higher than similar half-doped manganites such as Pr_0.5_Sr_0.5_MnO_3_ (~1.17 eV) [72] and Nd_0.5_Sr_0.5_MnO_3_ (~0.47 eV) [73], highlighting their distinct optical properties. The smaller ionic radius of Mg^2+^ (0.89 Å) compared to Ca^2+^ (1.18 Å) and Sr^2+^ (1.31 Å) induces lattice contraction, which modifies the average A-site cationic radius ❬r_A_❭ and alters the Mn–O–Mn bond angles. This structural distortion enhances crystal field effects and Jahn–Teller distortions, reducing the orbital overlap and decreasing the electronic bandwidth (W). The resulting distortion of the MnO_6_ octahedra further disrupts the electronic structure, influencing charge carrier dynamics and orbital hybridization. Consequently, these effects shift electronic states, increase carrier localization, and significantly widen the bandgap (3.25 eV for LCMMO, 3.71 eV for PCMMO) compared to Ca/Sr-doped systems. These characteristics suggest potential applicability in magnetic semiconductor devices [46]. These materials exhibit wide optical energy gaps (Eg > 3 eV), indicating potential suitability for various optoelectronic applications, such as gas sensors [74], solar cells [75], optoelectronic devices [76,77,78], liquid crystal displays (LCDs), and organic light-emitting diodes (OLEDs) [79,80,81,82]. However, further electrical conductivity measurements are necessary to fully assess their viability as transparent conductive oxides (TCOs), which will be explored in future studies. In these synthesized perovskite LCMMO and PCMMO nanoparticles, Mn cations exhibit a mixed valence state (Mn^3^⁺ and Mn^4^⁺), each surrounded by six oxygen anions forming MnO_6_ octahedral structures. Under the octahedral crystal field, the five 3d orbitals of the Mn cation split into low-lying $t_{2}g$ triplet and high-lying $e_{g}$ doublet sub-bands. For the Mn^4^⁺ ion (3d^3^ configuration), the $e_{g}\;$orbitals remain empty, whereas for Mn^3^⁺ (3d^4^ configuration), these orbitals contain one electron with spin aligned parallel to the core spin. Due to exchange interactions, the $e_{g}\;$sub-bands split further into up-spin and down-spin bands. The observed optical bandgaps are attributed to electronic transitions from the up-spin $e_{g}\;$band of Mn^3^⁺ (split by the Jahn–Teller effect) to the down-spin $e_{g}\;$band of a neighboring Mn^4^⁺ ion. The energy gap between these two $e_{g}$ bands correspond to Hund’s coupling energy (E_J_), as similarly reported for (La_0.6_Pr_0.4_)_0.65_Ca_0.35_MnO_3_ nanocrystals [46,80]. Given the wide distribution of the up-spin and down-spin $e_{g}\;$bands, excitation energy can extend into higher photon energy ranges.

**Figure 8:**
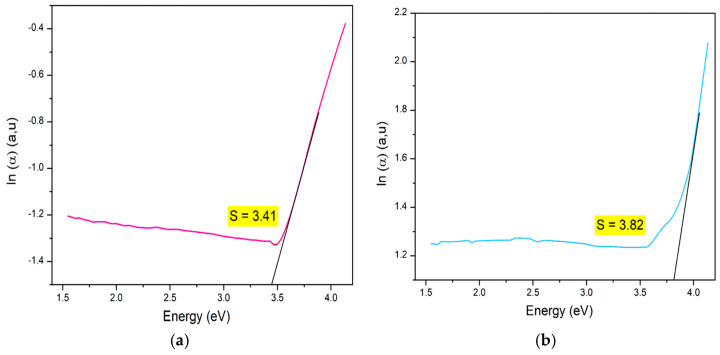
Urbach energy of (**a**) LCMMO and (**b**) PCMMO.

Determining the Urbach tail energy (E_u_) is essential for identifying the structural defects and vacancies that introduce localized states within the bandgap, especially in disordered or amorphous materials. Known as the band tail energy, E_u_ is critical for assessing crystallinity, structural disorder, and material imperfections [80]. E_u_ values are extracted from UV-Vis diffuse reflectance data, with higher Eu values indicating reduced crystallinity and increased disorder, leading to localized states extending into the bandgap. E_u_ quantifies the extent of localized states near the conduction band edge, revealing imperfections often arising from oxygen and magnetic ions as well as phonon-related disorder. Practically, E_u_ can be determined using the Urbach–Martienssen law [82]. For this study, Eu was determined by plotting ln(α) against hν and calculating its value through linear fitting, as shown in Figure 8a,b for LCMMO and PCMMO, respectively. The resulting slopes, approximately 3.4 and 3.8, correspond to the Urbach energies of E_u_ = 0.29 eV for LCMMO and E_u_ = 0.26 eV for PCMMO nanoceramics. These relatively low Urbach energy values suggest minimal structural disorder, consistent with high-quality perovskite oxides such as LSNMT ceramics (0.227 eV) [83], and LYCMO (0.2 eV) [79], which exhibit similar defect-suppressed behavior.

While the direct bandgaps of LCMMO and PCMMO suggest promise for ultraviolet (UV)-selective applications, the significant Urbach energies (3.41 eV and 3.82 eV) point to high defect densities and substantial structural disorder. These features likely limit the performance of the materials in applications such as high-efficiency optoelectronic devices or solar absorbers. For practical implementation, further optimization of synthesis to reduce disorder is essential. Therefore, the proposed applications must be considered with caution, and the current results are more appropriate for fundamental characterization or proof-of-concept systems.

## 5. Conclusions

In conclusion, this study successfully synthesized two novel perovskite nanoceramics, La_0.5_Ca_0.25_Mg_0.25_MnO_3_ (LCMMO) and Pr_0.5_Ca_0.25_Mg_0.25_MnO_3_ (PCMMO) using an environmentally friendly sol–gel method. This approach proved highly effective, yielding 100% of the desired nanoparticles. Comprehensive characterization of these materials was performed using a variety of techniques. Particle size analysis employed both the Williamson–Hall and Scherrer methods, providing complementary insights into the nanostructure. The chemical composition, homogeneity, and optical properties were confirmed through a combination of scanning electron microscopy (SEM), UV-vis absorbance spectroscopy, and Fourier-transform infrared (FTIR) spectroscopy. Notably, LCMMO and PCMMO exhibited distinct optical band gaps of 3.25 eV and 3.71 eV, respectively, highlighting their unique optical properties, potentially suitable for optoelectronics, like advanced materials [75,76]. The low Urbach energy values observed suggest minimal disorder or imperfections within the samples, indicating high-quality synthesis. The successful synthesis and thorough characterization of these novel nanoceramics not only expand our understanding of doped manganite perovskites but also pave the way for their potential applications in advanced materials science and technology. Future research could explore the performance of these materials in specific device configurations and investigate potential enhancements through further compositional tuning or processing optimizations.

## Figures and Tables

**Table 1:** Atomic Percentages (At. %) from XRF analysis.

At. %	La	Pr	Ca	Mg	Mn
LCMMO	4.44	/	2.75	2.10	7.31
PCMMO	**/**	7.38	4.36	3.83	13.69

**Table 2:** Crystallite size (D), diffraction angle (θ), and full width at half maximum (FWHM (β)) of both LCMMO and PCMMO nanoparticles.

PCMMO	Pos. [°2Th.]	32.843	40.4961	47.095	58.585
	FWHM (β) [°2Th.]	0.2047	0.2558	0.2578	0.3581
	D (nm)	42.00	33.09	33.87	25.43
	Dmoy (nm)	32.59			
LCMMO	Pos. [°2Th.]	33.056	40.762	47.419	58.993
	FWHM (β) [°2Th.]	0.30708	0.3581	0.5628	0.6140
	D (nm)	27.8	23.66	15.41	14.86
	Dmoy (nm)	20.43			

## Data Availability

Data are available upon reasonable request from the corresponding author.

## Supplementary Materials

The following supporting information can be downloaded at https://www.mdpi.com/article/10.3390/nano15070561/s1, Figure S1: Predicted crystalline structure distribution of perovskite manganites; Table S1: Previous studies on A-site-substituted perovskite manganites: comprehensive table. Table S2: Ionic radii of selected elements for perovskite manganites tolerance factor calculations (Shannon’s 9-Coordination Data). Table S3. Predicting crystal structure and tolerance factor in A-site-substituted perovskite manganites using Shannon’s ionic radius table.
