# Supplementary material for: Unlocking the Potential of Mg-Doped Rare Earth Manganites: Machine Learning-Guided Synthesis and Insights into Structural and Optical Properties
Source: Nanomaterials (Basel). 2025 Apr 6;15(7):561. doi: 10.3390/nano15070561 (PMC11990812; doi:10.3390/nano15070561)
Supplement: Supplementary file 1 [file nanomaterials-15-00561-s001.zip › nanomaterials-3502239-supplementary.pdf]

Supporting Information for:

Unlocking the Potential of Mg-Doped Rare Earth Manganites: Machine Learning-Guided Synthesis and Insights into Structural and Optical Properties

Table S1. Previous Studies On A-Site Substituted Perovskite Manganites: Comprehensive Table.

	**1/- Simple : $Re_{1-x}Ae_xMnO_3$**			**2/- Re+2Ae: $Re_{1-x}(Ae_{1-y}+Ae_y)_xMnO_3$**		
	**Mg**	**Ba**	**Ca**	**Ca+Mg**	**Ca+Ba**	**Ba+Mg**
**Ce**	$Ce_{1-x}Mg_xMnO_3$	$Ce_{1-x}Ba_xMnO_3$	$Ce_{1-x}Ca_xMnO_3$ [1-21]	$Ce_{1-x}(Ca_{1-y}Mg_y)_xMnO_3$	$Ce_{1-x}(Ca_{1-y}Ba_y)_xMnO_3$	$Ce_{1-x}(Ba_{1-y}Mg_y)_xMnO_3$
**Pr**	$Pr_{1-x}Mg_xMnO_3$	$Pr_{1-x}Ba_xMnO_3$ [24-54]	$Pr_{1-x}Ca_xMnO_3$	**$Pr_{1-x}(Ca_{1-y}Mg_y)_xMnO_3$** ✓	$Pr_{1-x}(Ca_{1-y}Ba_y)_xMnO_3$ [55-61]	$Pr_{1-x}(Ba_{1-y}Mg_y)_xMnO_3$
**Gd**	$Gd_{1-x}Mg_xMnO_3$	$Gd_{1-x}Ba_xMnO_3$ [62-74]	$Gd_{1-x}Ca_xMnO_3$ [75-97]	$Gd_{1-x}(Ca_{1-y}Mg_y)_xMnO_3$	$Gd_{1-x}(Ca_{1-y}Ba_y)_xMnO_3$	$Gd_{1-x}(Ba_{1-y}Mg_y)_xMnO_3$
**Ho**	$Ho_{1-x}Mg_xMnO_3$	$Ho_{1-x}Ba_xMnO_3$	$Ho_{1-x}Ca_xMnO_3$ [98-115]	$Ho_{1-x}(Ca_{1-y}Mg_y)_xMnO_3$	$Ho_{1-x}(Ca_{1-y}Ba_y)_xMnO_3$	$Ho_{1-x}(Ba_{1-y}Mg_y)_xMnO_3$
**La**	$La_{1-x}Mg_xMnO_3$ [129-139]	$La_{1-x}Ba_xMnO_3$	$La_{1-x}Ca_xMnO_3$	**$La_{1-x}(Ca_{1-y}Mg_y)_xMnO_3$** ✓ [140]	$La_{1-x}(Ca_{1-y}Ba_y)_xMnO_3$ [141-150]	$La_{1-x}(Ba_{1-y}Mg_y)_xMnO_3$

	**3/- 2Re+Ae : $(Re_{1-y}+Re_y)_{1-x}Ae_xMnO_3$**		
	**Mg**	**Ba**	**Ca**
**Ce + Pr**	$(Ce_{1-y}Pr_y)_{1-x}Mg_xMnO_3$	$(Ce_{1-y}Pr_y)_{1-x}Ba_xMnO_3$	$(Ce_{1-y}Pr_y)_{1-x}Ca_xMnO_3$ [22-23]
**Ce + Gd**	$(Ce_{1-y}Gd_y)_{1-x}Mg_xMnO_3$	$(Ce_{1-y}Gd_y)_{1-x}Ba_xMnO_3$	$(Ce_{1-y}Gd_y)_{1-x}Ca_xMnO_3$
**Ce + Ho**	$(Ce_{1-y}Ho_y)_{1-x}Mg_xMnO_3$	$(Ce_{1-y}Ho_y)_{1-x}Ba_{1-x}MnO_3$	$(Ce_{1-y}Ho_y)_{1-x}Ca_xMnO_3$
**Ce + La**	$(Ce_{1-y}La_y)_{1-x}Mg_xMnO_3$	$(Ce_{1-y}La_y)_{1-x}Ba_{1-x}MnO_3$ [116]	$(Ce_{1-y}La_y)_{1-x}Ca_xMnO_3$ [117-128]
**Pr + Gd**	$(Pr_{1-y}Gd_y)_{1-x}Mg_xMnO_3$	$(Pr_{1-y}Gd_y)_{1-x}Ba_{1-x}MnO_3$	$(Pr_{1-y}Gd_y)_{1-x}Ca_xMnO_3$ [151-152]
**Pr + Ho**	$(Pr_{1-y}Ho_y)_{1-x}Mg_xMnO_3$	$(Pr_{1-y}Ho_y)_{1-x}Ba_{1-x}MnO_3$	$(Pr_{1-y}Ho_y)_{1-x}Ca_xMnO_3$
**Pr + La**	$(Pr_{1-y}La_y)_{1-x}Mg_xMnO_3$	$(Pr_{1-y}La_y)_{1-x}Ba_{1-x}MnO_3$ [153-161]	$(Pr_{1-y}La_y)_{1-x}Ca_xMnO_3$
**Gd + Ho**	$(Gd_{1-y}Ho_y)_{1-x}Mg_xMnO_3$	$(Gd_{1-y}Ho_y)_{1-x}Ba_{1-x}MnO_3$	$(Gd_{1-y}Ho_y)_{1-x}Ca_xMnO_3$
**Gd + La**	$(Gd_{1-y}La_y)_{1-x}Mg_xMnO_3$	$(Gd_{1-y}La_y)_{1-x}Ba_xMnO_3$ [162-163]	$(Gd_{1-y}La_y)_{1-x}Ca_xMnO_3$ [164-171]
**Ho + La**	$(Ho_{1-y}La_y)_{1-x}Mg_xMnO_3$	$(Ho_{1-y}La_y)_{1-x}Ba_xMnO_3$	$(Ho_{1-y}La_y)_{1-x}Ca_xMnO_3$ [172-173]

Table S2. Ionic Radii of Selected Elements for Perovskite Manganites Tolerance Factor Calculations (Shannon's 9-Coordination Data) [174].

Element	La	Ce	Pr	Gd	Ho	Ba	Ca	Mg	Mn^{3+}	Mn4+	O
**Ionic Radius (A°)**	1.216	1.196	1.179	1.107	1.072	1.47	1.18	0.89	0.645	0.53	1.35

$$\boldsymbol{t} = \frac{r_A + r_O}{\sqrt{2}(r_B + r_O)} \qquad \textbf{Eq .S1}$$

Where:

$\boldsymbol{t}$**:** Tolerance factor, introduced by Goldschmidt.

$\boldsymbol{r_A}$**:** Ionic radius of the A-site cation (large cation) in perovskites: La^{3+}, Ce^{3+}, Pr^{3+}, Gd^{3+}, Ho^{3+}, Ba^{2+}, Ca^{2+}, Mg^{2+}.

$\boldsymbol{r_B}$**:** Ionic radius of the B-site cation (small cation) in perovskites: Mn^{3+}/ Mn^{4+}.

$\boldsymbol{r_O}$**:** Ionic radius of Oxygen anion.

Determining the ionic radius of site A in a perovskite structure when dealing with a solid solution composed of more than two elements:

$$\langle r_A \rangle = (1\text{-}x)\,((1\text{-}y)\, r_{A1} + y r_{A2}) + x r_{A3} \qquad \textbf{Eq .S2}$$

Example (in A-Site: $Pr_{0.5}Ca_{0.25}Mg_{0.25}$):

$$\langle r_{\mathrm{Pr0.5Ca0.25Mg0.25}} \rangle = 0.5\, r_{Pr} + 0.25\, r_{Ca} + 0.25\, r_{Mg} \qquad \textbf{Eq .S3}$$

Table S3. Predicting Crystal Structure and Tolerance Factor in A-Site Substituted Perovskite Manganites Using Shannon's Ionic Radius Table [174].

	1/- $Re_{0.5}Ae_{0.5}MnO_3$			2/- $Re_{0.5}(Ae_{0.25}+Ae_{0.25})MnO_3$		
	**Mg**	**Ba**	**Ca**	**(Ca,Mg)**	**(Ca,Ba)**	**(Ba,Mg)**
**Ce**	$Ce_{0.5}Mg_{0.5}MnO_3$<br>**t=0.87**<br>**O**	$Ce_{0.5}Ba_{0.5}MnO_3$<br>**t=0.98**<br>**R**	$Ce_{0.5}Ca_{0.5}MnO_3$<br>**t=0.93**<br>**O**	$Ce_{0.5}Ca_{0.25}Mg_{0.25}MnO_3$<br>**t=0.9**<br>**O**	$Ce_{0.5}Ca_{0.25}Ba_{0.25}MnO_3$<br>**t=0.953**<br>**R**	$Ce_{0.5}Ba_{0.25}Mg_{0.25}MnO_3$<br>**t=0.926**<br>**O**
**Pr**	$Pr_{0.5}Mg_{0.5}MnO_3$<br>**t=0.87**<br>**O**	$Pr_{0.5}Ba_{0.5}MnO_3$<br>**t=0.97**<br>**R**	$Pr_{0.5}Ca_{0.5}MnO_3$<br>**t=0.92**<br>**O**	**$Pr_{0.5}Ca_{0.25}Mg_{0.25}MnO_3$**<br>**t=0.897**<br>**O**	$Pr_{0.5}Ca_{0.25}Ba_{0.25}MnO_3$<br>**t=0.95**<br>**R**	$Pr_{0.5}Ba_{0.25}Mg_{0.25}MnO_3$<br>**t=0.923**<br>**O**
**Gd**	$Gd_{0.5}Mg_{0.5}MnO_3$<br>**t=0.86**<br>**O**	$Gd_{0.5}Ba_{0.5}MnO_3$<br>**t=0.96**<br>**R**	$Gd_{0.5}Ca_{0.5}MnO_3$<br>**t=0.91**<br>**O**	$Gd_{0.5}Ca_{0.25}Mg_{0.25}MnO_3$<br>**t=0.884**<br>**O**	$Gd_{0.5}Ca_{0.25}Ba_{0.25}MnO_3$<br>**t=0.936**<br>**O**	$Gd_{0.5}Ba_{0.25}Mg_{0.25}MnO_3$<br>**t=0.91**<br>**O**
**Ho**	$Ho_{0.5}Mg_{0.5}MnO_3$<br>**t=0.85**<br>**O**	$Ho_{0.5}Ba_{0.5}MnO_3$<br>**t=0.96**<br>**R**	$Ho_{0.5}Ca_{0.5}MnO_3$<br>**t=0.9**<br>**O**	$Ho_{0.5}Ca_{0.25}Mg_{0.25}MnO_3$<br>**t=0.877**<br>**O**	$Ho_{0.5}Ca_{0.25}Ba_{0.25}MnO_3$<br>**t=0.93**<br>**O**	$Ho_{0.5}Ba_{0.25}Mg_{0.25}MnO_3$<br>**t=0.904**<br>**O**
**La**	$La_{0.5}Mg_{0.5}MnO_3$<br>**t=0.88**<br>**O**	$La_{0.5}Ba_{0.5}MnO_3$<br>**t=0.98**<br>**R**	$La_{0.5}Ca_{0.5}MnO_3$<br>**t=0.93**<br>**O**	**$La_{0.5}Ca_{0.25}Mg_{0.25}MnO_3$**<br>**t=0.903**<br>**O**	$La_{0.5}Ca_{0.25}Ba_{0.25}MnO_3$<br>**t=0.956**<br>**R**	$La_{0.5}Ba_{0.25}Mg_{0.25}MnO_3$<br>**t=0.93**<br>**O**

	3/- $(Re_{0.25}+Re_{0.25})Ae_{0.5}MnO_3$		
	**Mg**	**Ba**	**Ca**
**Ce + Pr**	$Ce_{0.25}Pr_{0.25}Mg_{0.5}MnO_3$<br>**t=0.872**<br>**O**	$Ce_{0.25}Pr_{0.25}Ba_{0.5}MnO_3$<br>**t=0.978**<br>**R**	$Ce_{0.25}Pr_{0.25}Ca_{0.5}MnO_3$<br>**t=0.925**<br>**O**
**Ce + Gd**	$Ce_{0.25}Gd_{0.25}Mg_{0.5}MnO_3$<br>**t=0.865**<br>**O**	$Ce_{0.25}Gd_{0.25}Ba_{0.5}MnO_3$<br>**t=0.971**<br>**R**	$Ce_{0.25}Gd_{0.25}Ca_{0.5}MnO_3$<br>**t=0.918**<br>**O**
**Ce + Ho**	$Ce_{0.25}Ho_{0.25}Mg_{0.5}MnO_3$<br>**t=0.862**<br>**O**	$Ce_{0.25}Ho_{0.25}Ba_{0.5}MnO_3$<br>**t=0.968**<br>**R**	$Ce_{0.25}Ho_{0.25}Ca_{0.5}MnO_3$<br>**t=0.915**<br>**O**
**Ce + La**	$Ce_{0.25}La_{0.25}Mg_{0.5}MnO_3$<br>**t=0.875**<br>**O**	$Ce_{0.25}La_{0.25}Ba_{0.5}MnO_3$<br>**t=0.981**<br>**R**	$Ce_{0.25}La_{0.25}Ca_{0.5}MnO_3$<br>**t=0.928**<br>**O**
**Pr + Gd**	$Pr_{0.25}Gd_{0.25}Mg_{0.5}MnO_3$<br>**t=0.864**<br>**O**	$Pr_{0.25}Gd_{0.25}Ba_{0.5}MnO_3$<br>**t=0.97**<br>**R**	$Pr_{0.25}Gd_{0.25}Ca_{0.5}MnO_3$<br>**t=0.917**<br>**O**
**Pr + Ho**	$Pr_{0.25}Ho_{0.25}Mg_{0.5}MnO_3$<br>**t=0.86**<br>**O**	$Pr_{0.25}Ho_{0.25}Ba_{0.5}MnO_3$<br>**t=0.966**<br>**R**	$Pr_{0.25}Ho_{0.25}Ca_{0.5}MnO_3$<br>**t=0.913**<br>**O**
**Pr + La**	$Pr_{0.25}La_{0.25}Mg_{0.5}MnO_3$<br>**t=0.874**<br>**O**	$Pr_{0.25}La_{0.25}Ba_{0.5}MnO_3$<br>**t=0.979**<br>**R**	$Pr_{0.25}La_{0.25}Ca_{0.5}MnO_3$<br>**t=0.93**<br>**O**
**Gd + Ho**	$Gd_{0.25}Ho_{0.25}Mg_{0.5}MnO_3$<br>**t=0.854**<br>**O**	$Gd_{0.25}Ho_{0.25}Ba_{0.5}MnO_3$<br>**t=0.96**<br>**R**	$Gd_{0.25}Ho_{0.25}Ca_{0.5}MnO_3$<br>**t=0.907**<br>**O**
**Gd + La**	$Gd_{0.25}La_{0.25}Mg_{0.5}MnO_3$<br>**t=0.867**<br>**O**	$Gd_{0.25}La_{0.25}Ba_{0.5}MnO_3$<br>**t=0.973**<br>**R**	$Gd_{0.25}La_{0.25}Ca_{0.5}MnO_3$<br>**t=0.92**<br>**O**
**Ho + La**	$Ho_{0.25}La_{0.25}Mg_{0.5}MnO_3$<br>**t=0.864**<br>**O**	$Ho_{0.25}La_{0.25}Ba_{0.5}MnO_3$<br>**0.97**<br>**R**	$Ho_{0.25}La_{0.25}Ca_{0.5}MnO_3$<br>**t=0.917**<br>**O**

Notes:

- Re: Rare earth element
- Ae: Alkaline earth element
- t: Tolerance factor
- O: Orthorhombic structure
- R: Rhombohedral structure

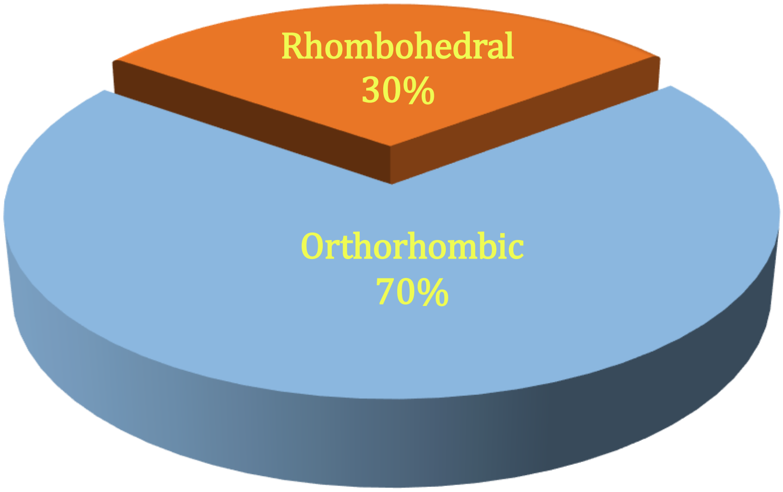

Figure S1. Predicted Crystalline Structure Distribution of Perovskite Manganites

References

[1] A. Sharma, S. Sarangi, and S. V. Bhat, “Anomalous spin dynamics in the charge-ordered two-electron doped manganite Ca 0.9 Ce 0.1 Mn O 3 : Possibility of a spin-liquid phase,” *Phys. Rev. B*, vol. 73, no. 3, p. 035129, Jan. 2006, doi: 10.1103/PhysRevB.73.035129.

[2] E. N. Caspi *et al.*, “Structural and magnetic phase diagram of the two-electron-doped (Ca 1 − x Ce x) MnO 3 system: Effects of competition among charge, orbital, and spin ordering,” *Phys. Rev. B*, vol. 69, no. 10, p. 104402, Mar. 2004, doi: 10.1103/PhysRevB.69.104402.

[3] A. Maignan, C. Martin, F. Damay, and B. Raveau, “Factors Governing the Magnetoresistance Properties of the Electron-Doped Manganites $Ca_{1-x}A_{x}MnO_3$ (A = Ln, Th),” *Chem. Mater.*, vol. 10, no. 4, pp. 950–954, Apr. 1998, doi: 10.1021/cm970781b.

[4] M. Matsukawa, A. Tamura, Y. Yamato, T. Kumagai, S. Nimori, and R. Suryanarayanan, “Thermal transport in electron-doped manganites Ca1–xCexMnO3,” *Journal of Magnetism and Magnetic Materials*, vol. 310, no. 2, pp. e283–e285, 2007.

[5] Y. Yamato, M. Matsukawa, Y. Murano, S. Kobayashi, and R. Suryanarayanan, “Current-induced nonlinear conduction of two-electron doped manganites Ca1- xCexMnO3,” *Journal of Physics D: Applied Physics*, vol. 43, no. 14, p. 145003, 2010.

[6] J. Dukić, S. Bošković, and B. Matović, “Crystal structure of Ce-doped CaMnO3 perovskite,” *Ceramics International*, vol. 35, no. 2, pp. 787–790, 2009.

[7] A. Srivastava and N. K. Gaur, “The role of lattice distortions in determining the thermal properties of electron doped CaMnO3,” *Journal of Physics: Condensed Matter*, vol. 21, no. 9, p. 096001, 2009.

[8] B. Hong *et al.*, “The coupling interaction between charge ordering and spin ordering in a CO/C-type AFM system,” *Solid state communications*, vol. 144, no. 1–2, pp. 31–36, 2007.
[9] B. J. Kennedy, J. Ting, Q. Zhou, Z. Zhang, M. Matsuda, and M. Miyake, “Structural characterisation of the perovskite series Sr0. 9- xCaxCe0. 1MnO3: Influence of the Jahn–Teller effect,” *Journal of Solid State Chemistry*, vol. 182, no. 4, pp. 954–959, 2009.
[10] Y. Wang, Y. Sui, X. Wang, and W. Su, “Structure, transport and magnetic properties of electron-doped perovskites RxCa1- xMnO3 (R= La, Y and Ce),” *Journal of Physics: Condensed Matter*, vol. 21, no. 19, p. 196004, 2009.
[11] C. Lucas *et al.*, “Preparation and characterization of Ca1 − x Ce x MnO3 perovskite electrodes,” *J Solid State Electrochem*, vol. 13, no. 6, pp. 943–950, Jun. 2009, doi: 10.1007/s10008-008-0630-3.
[12] T. Harano *et al.*, “Phase diagram of Ca1- xCexMnO3 thin films studied by X-ray magnetic circular dichroism,” *Solid state communications*, vol. 174, pp. 30–33, 2013.
[13] Y. Wang, Y. Sui, X. Wang, and W. Su, “Effects of substituting La3+, Y3+ and Ce4+ for Ca2+ on the high temperature transport and thermoelectric properties of CaMnO3,” *Journal of Physics D: Applied Physics*, vol. 42, no. 5, p. 055010, 2009.
[14] N. Imamura, T. Mizoguchi, H. Yamauchi, and M. Karppinen, “Multivariate data analysis approach to understand magnetic properties of perovskite manganese oxides,” *Journal of Solid State Chemistry*, vol. 181, no. 5, pp. 1195–1203, 2008.
[15] N. N. Loshkareva, E. G. Gerasimov, N. V. Mushnikov, A. V. Korolyov, and A. M. Balbashov, “Metamagnetic transitions in electron-doped single crystals of manganites Ca1- x (Ln) xMnO3,(Ln= La, Ce; x≤ 0.12),” *Journal of Physics: Condensed Matter*, vol. 22, no. 35, p. 356003, 2010.
[16] N. N. Loshkareva *et al.*, “Magnetic, electrical, and optical properties of Ca1 − x Ce x MnO3 (x ≤ 0.12) single crystals,” *J. Exp. Theor. Phys.*, vol. 108, no. 1, pp. 88–97, Jan. 2009, doi: 10.1134/S1063776109010129.
[17] D. Varshney and I. Mansuri, “Influence of Ce Doping on Structural and Transport Properties of Ca1−x Ce x MnO3 (x=0.2) Manganite,” *J Low Temp Phys*, vol. 162, no. 1–2, pp. 52–61, Jan. 2011, doi: 10.1007/s10909-010-0233-0.
[18] Y. Wang *et al.*, “High Temperature Thermoelectric Response of Electron-Doped $CaMnO_3$,” *Chem. Mater.*, vol. 21, no. 19, pp. 4653–4660, Oct. 2009, doi: 10.1021/cm901766y.
[19] W. J. Lu, Y. P. Sun, B. C. Zhao, X. B. Zhu, and W. H. Song, “Giant electroresistance and nonlinear conduction in electron-doped Ca0. 9Ce0. 1MnO3,” *Solid state communications*, vol. 137, no. 6, pp. 288–291, 2006.
[20] N. N. Loshkareva and E. V. Mostovshchikova, “Electron-doped manganites based on CaMnO3,” *Phys. Metals Metallogr.*, vol. 113, no. 1, pp. 19–38, Jan. 2012, doi: 10.1134/S0031918X12010073.
[21] R. Ganguly, M. Hervieu, A. Maignan, C. Martin, and B. Raveau, “Doping at the Mn site of the electron-doped manganite Ca0. 9Ce0. 1MnO3,” *Journal of Physics: Condensed Matter*, vol. 14, no. 39, p. 9039, 2002.
[22] K. R. Mavani, A. D. Hillier, P. L. Paulose, W. A. Kockelmann, and D. T. Adroja, “Spin dynamics of (Pr0. 5-xCex) Ca0. 5MnO3 (x= 0.05, 0.10, and 0.20) system studied by muon spin relaxation,” *Journal of Applied Physics*, vol. 112, no. 7, 2012, Accessed: Dec. 10, 2024. [Online]. Available: https://pubs.aip.org/aip/jap/article/112/7/073911/369525

[23] K. R. Mavani and P. L. Paulose, "Influence of Ce substitution on structure, magnetism, and transport in Pr 0.5 − x Ce x Ca 0.5 MnO 3 (0.03 ⩽ x ⩽ 0.20) : From metamagnetism to a spin-glass state," *Phys. Rev. B*, vol. 72, no. 10, p. 104421, Sep. 2005, doi: 10.1103/PhysRevB.72.104421.
[24] M. Ellouze, W. Boujelben, A. Cheikhrouhou, H. Fuess, and R. Madar, "Vacancy effects on the crystallographic and magnetic properties in lacunar Pr0. 7Ba0. 3- xMnO3 oxides," *Solid state communications*, vol. 124, no. 4, pp. 125–130, 2002.
[25] V. Sen, N. Panwar, G. L. Bhalla, and S. K. Agarwal, "Structural, electrical and magnetic properties of Sb-doped Pr2/3Ba1/3MnO3 perovskite manganites," *Journal of alloys and compounds*, vol. 439, no. 1–2, pp. 205–209, 2007.
[26] M. Ellouze, W. Boujelben, A. Cheikhrouhou, H. Fuess, and R. Madar, "Structure, ferromagnetism and magnetotransport properties in the barium-deficient Pr0. 7Ba0. 2□ 0.1 MnO3 manganite oxides," *Journal of magnetism and magnetic materials*, vol. 257, no. 2–3, pp. 319–326, 2003.
[27] I. O. Troyanchuk, S. V. Trukhanov, H. Szymczak, and K. Baerner, "Effect of oxygen content on the magnetic and transport properties of Pr0. 5Ba0. 5MnO3-γ," *Journal of Physics: Condensed Matter*, vol. 12, no. 7, p. L155, 2000.
[28] D. C. Krishna and P. V. Reddy, "Magnetic transport behavior of nano-crystalline Pr0. 67A0. 33MnO3 (A= Ca, Sr, Pb and Ba) manganites," *Journal of alloys and compounds*, vol. 479, no. 1–2, pp. 661–669, 2009.
[29] N. Imamura, T. Mizoguchi, H. Yamauchi, and M. Karppinen, "Multivariate data analysis approach to understand magnetic properties of perovskite manganese oxides," *Journal of Solid State Chemistry*, vol. 181, no. 5, pp. 1195–1203, 2008.
[30] A. Muñoz, J. A. Alonso, M. J. Martınez-Lope, and M. T. Fernández-Dıaz, "Magnetic structure evolution of Pr1- xMnO3 perovskite from neutron powder diffraction data," *Solid state communications*, vol. 113, no. 4, pp. 227–231, 1999.
[31] P. K. Siwach, H. K. Singh, and O. N. Srivastava, "Low field magnetotransport in manganites," *Journal of Physics: Condensed Matter*, vol. 20, no. 27, p. 273201, 2008.
[32] A. Barnabé, *et al.*, "Barium-Based Manganites Ln_{1-x} Ba_x MnO_3 with Ln = {Pr, La}: Phase Transitions and Magnetoresistance Properties," *Chem. Mater.*, vol. 10, no. 1, pp. 252–259, Jan. 1998, doi: 10.1021/cm9704084.
[33] S. V. Trukhanov, I. O. Troyanchuk, M. Hervieu, H. Szymczak, and K. Bärner, "Magnetic and electrical properties of L BaMn 2 O 6 − γ (L = Pr , Nd, Sm, Eu, Gd, Tb) manganites," *Phys. Rev. B*, vol. 66, no. 18, p. 184424, Nov. 2002, doi: 10.1103/PhysRevB.66.184424.
[34] A. K. Kundu, M. M. Seikh, K. Ramesha, and C. N. R. Rao, "Novel effects of size disorder on the electronic and magnetic properties of rare earth manganates of the type La0. 7- xLnxBa0. 3MnO3 (Ln= Pr, Nd, Gd or Dy) with large average radius of the A-site cations," *Journal of Physics: Condensed Matter*, vol. 17, no. 26, p. 4171, 2005.
[35] A. Klimkowicz, K. Świerczek, A. Takasaki, J. Molenda, and B. Dabrowski, "Crystal structure and oxygen storage properties of BaLnMn2O5+ δ (Ln: Pr, Nd, Sm, Gd, Dy, Er and Y) oxides," *Materials Research Bulletin*, vol. 65, pp. 116–122, 2015.
[36] S. V. Trukhanov *et al.*, "Crystal structure and magnetic properties of Ba-ordered manganites Ln0.70Ba0.30MnO3−δ (Ln = Pr, Nd)," *J. Exp. Theor. Phys.*, vol. 103, no. 3, pp. 398–410, Sep. 2006, doi: 10.1134/S1063776106090093.
[37] I. O. Troyanchuk, I. M. Kolesova, H. Szymczak, and A. Nabialek, "Preparation, magnetic and transport properties of A0. 66Ba0. 34MnO3- y (A= Pr, Nd, Sm, Eu, Gd) perovskites," *Journal of magnetism and magnetic materials*, vol. 176, no. 2–3, pp. 267–271, 1997.

[38] E. Pollert, "Influence of Mn3+ ions on ordering in magnetic oxides," *International Journal of Inorganic Materials*, vol. 2, no. 6, pp. 661–670, 2000.
[39] G. Venkataiah, Y. K. Lakshmi, and P. V. Reddy, "Thermopower studies of Pr0. 67D0. 33MnO3 manganite system," *Journal of Physics D: Applied Physics*, vol. 40, no. 3, p. 721, 2007.
[40] S. Panwar, V. Kumar, A. Chaudhary, R. Kumar, and I. Singh, "Theoretical study of magnetotransport properties of colossal magnetoresistive manganites (Re1- xAxMnO3): A variational treatment," *Solid State Communications*, vol. 223, pp. 32–36, 2015.
[41] O. Yu. Gorbenko *et al.*, "Crystallographic, magnetic, and electrical properties of thin Re 0.6Ba0.4MnO3 epitaxial films (Re = La, Pr, Nd, Gd)," *Phys. Solid State*, vol. 46, no. 7, pp. 1255–1261, Jul. 2004, doi: 10.1134/1.1778450.
[42] T. Nakajima, H. Kageyama, H. Yoshizawa, K. Ohoyama, and Y. Ueda, "Ground State Properties of the *A* -site Ordered Manganites, *R* $BaMn_2$ O_6 (*R* = La, Pr and Nd)," *J. Phys. Soc. Jpn.*, vol. 72, no. 12, pp. 3237–3242, Dec. 2003, doi: 10.1143/JPSJ.72.3237.
[43] N. Tomohiko and U. Yutaka, "Structures and electromagnetic properties of the A-site disordered Ba-based manganites: R0. 5 Ba0. 5 MnO3 (R= Y and rare earth elements)," *Journal of Alloys and Compounds*, vol. 383, pp. 135–139, 2004.
[44] Y. Ueda and T. Nakajima, "Novel structures and electromagnetic properties of the A-site-ordered/disordered manganites RBaMn2O6/R0. 5Ba0. 5MnO3 (R= Y and rare earth elements)," *Journal of Physics: Condensed Matter*, vol. 16, no. 11, p. S573, 2004.
[45] A. M. Aliev, A. G. Gamzatov, A. B. Batdalov, V. S. Kalitka, and A. R. Kaul, "Direct and inverse magnetocaloric effects in A-site ordered PrBaMn2O6 manganite," *Journal of alloys and compounds*, vol. 509, no. 17, pp. L165–L167, 2011.
[46] T. Nakajima, H. Kageyama, and Y. Ueda, "Dramatic change of magnetic property in the A-site ordered/disordered manganites PrBaMn2O6/Pr0. 5Ba0. 5MnO3," *Journal of magnetism and magnetic materials*, vol. 272, pp. 405–406, 2004.
[47] S. V. Trukhanov *et al.*, "Study of A-site ordered PrBaMn2O6- δ manganite properties depending on the treatment conditions," *Journal of Physics: Condensed Matter*, vol. 17, no. 41, p. 6495, 2005.
[48] S. V. Trukhanov, I. O. Troyanchuk, I. M. Fita, H. Szymczak, and K. Bärner, "Comparative study of the magnetic and electrical properties of Pr1- xBaxMnO3- δ manganites depending on the preparation conditions," *Journal of magnetism and magnetic materials*, vol. 237, no. 3, pp. 276–282, 2001.
[49] S. V. Trukhanov, A. V. Trukhanov, C. E. Botez, A. H. Adair, H. Szymczak, and R. Szymczak, "Phase separation and size effects in Pr0. 70Ba0. 30MnO3+ δ perovskite manganites," *Journal of Physics: Condensed Matter*, vol. 19, no. 26, p. 266214, 2007.
[50] N. Panwar, I. Coondoo, R. S. Singh, and S. K. Agarwal, "Intrinsic and extrinsic transport properties of Pr0. 67Ba0. 33MnO3: Ag2O composites," *Journal of alloys and compounds*, vol. 507, no. 2, pp. 439–442, 2010.
[51] K. B. Garg *et al.*, "Study of Sb substitution for Pr in the Pr0. 67Ba0. 33MnO3 system," *Journal of magnetism and magnetic materials*, vol. 321, no. 4, pp. 305–311, 2009.
[52] S. Hcini, S. Zemni, A. Triki, H. Rahmouni, and M. Boudard, "Size mismatch, grain boundary and bandwidth effects on structural, magnetic and electrical properties of Pr0. 67Ba0. 33MnO3 and Pr0. 67Sr0. 33MnO3 perovskites," *Journal of Alloys and Compounds*, vol. 509, no. 5, pp. 1394–1400, 2011.

[53] D. C. Krishna and P. V. Reddy, "Magnetic transport behavior of nano-crystalline Pr0. 67A0. 33MnO3 (A= Ca, Sr, Pb and Ba) manganites," *Journal of alloys and compounds*, vol. 479, no. 1–2, pp. 661–669, 2009.

[54] W. Boujelben, A. Cheikh-Rouhou, M. Ellouze, and J. C. Joubert, "Vacancy effects on the physical properties in lacunar Pr0. 7- x□ xBa0. 3MnO3 oxides," *Journal of magnetism and magnetic materials*, vol. 242, pp. 662–664, 2002.

[55] T.-L. Phan, P. Zhang, T. D. Thanh, Q. T. Tran, and S. C. Yu, "Second-order magnetic phase transition in orthorhombic Pr0. 7Ca0. 15Ba0. 15MnO3," *Solid state communications*, vol. 191, pp. 25–29, 2014.

[56] A. N. Ulyanov, H. D. Quang, N. E. Pismenova, S. C. Yu, and G. G. Levchenko, "Pr0. 7Ca0. 15Ba0. 15MnO3 manganite: Electron paramagnetic resonance, conductivity and susceptibility," *Solid state communications*, vol. 152, no. 16, pp. 1556–1559, 2012.

[57] J. A. Collado, J. L. García-Muñoz, and M. A. G. Aranda, "Effects of the A-site cation number on the properties of Ln5/8M3/8MnO3 manganites," *Journal of Solid State Chemistry*, vol. 183, no. 5, pp. 1083–1089, 2010.

[58] D. Zhu, V. Hardy, A. Maignan, and B. Raveau, "Relationship between the onset of ferromagnetism and the training effect in CMR perovskite manganites," *Journal of Physics: Condensed Matter*, vol. 16, no. 8, p. L101, 2004.

[59] D. Zhu, B. Raveau, V. Hardy, A. Maignan, M. Hervieu, and C. Martin, "Ga and Ba substitution in charge ordered Pr1- xCaxMnO3: exceptional predisposition of the x= 0.43 compound to ferromagnetism," *Journal of Physics: Condensed Matter*, vol. 16, no. 16, p. 2861, 2004.

[60] D. Akahoshi, D. Iijima, and T. Saito, "The effect of Ba-site substitution on the magnetic behavior of ordered perovskite RBaMn2O6 (R= rare earth)," *Journal of Solid State Chemistry*, vol. 228, pp. 105–109, 2015.

[61] B. Raveau *et al.*, "Sharp magnetization steps induced by A-site substitution in Pr0. 5Ca0. 5MnO3," *Journal of Physics: Condensed Matter*, vol. 15, no. 41, p. 7055, 2003.

[62] I. O. Troyanchuk, S. V. Trukhanov, D. D. Khalyavin, and H. Szymczak, "Magnetic properties of anion deficit manganites Ln0. 55Ba0. 45MnO3- γ (Ln= La, Nd, Sm, Gd, γ⩽ 0.37)," *Journal of magnetism and magnetic materials*, vol. 208, no. 3, pp. 217–220, 2000.

[63] A. K. Kundu, P. Nordblad, and C. N. R. Rao, "Glassy behaviour of the ferromagnetic and the non-magnetic insulating states of the rare earth manganates Ln0. 7Ba0. 3MnO3 (Ln= Nd or Gd)," *Journal of Physics: Condensed Matter*, vol. 18, no. 20, p. 4809, 2006.

[64] S. V. Trukhanov, I. O. Troyanchuk, M. Hervieu, H. Szymczak, and K. Bärner, "Magnetic and electrical properties of L BaMn 2 O 6 − γ (L = Pr , Nd, Sm, Eu, Gd, Tb) manganites," *Phys. Rev. B*, vol. 66, no. 18, p. 184424, Nov. 2002, doi: 10.1103/PhysRevB.66.184424.

[65] A. K. Kundu, M. M. Seikh, K. Ramesha, and C. N. R. Rao, "Novel effects of size disorder on the electronic and magnetic properties of rare earth manganates of the type La0. 7- xLnxBa0. 3MnO3 (Ln= Pr, Nd, Gd or Dy) with large average radius of the A-site cations," *Journal of Physics: Condensed Matter*, vol. 17, no. 26, p. 4171, 2005.

[66] A. A. Taskin, A. N. Lavrov, and Y. Ando, "Fast oxygen diffusion in A-site ordered perovskites," *Progress in Solid State Chemistry*, vol. 35, no. 2–4, pp. 481–490, 2007.

[67] H. Dai *et al.*, "Effect of barium doping on the microstructure, dielectric and magnetic properties of GdMnO3 multiferroic ceramics," *J Mater Sci: Mater Electron*, vol. 30, no. 3, pp. 2523–2529, Feb. 2019, doi: 10.1007/s10854-018-0526-7.

[68] A. Klimkowicz, K. Świerczek, T. Rząsa, A. Takasaki, and B. Dabrowski, "Oxygen storage properties and catalytic activity of layer-ordered perovskites BaY1- xGdxMn2O5+ δ," *Solid State Ionics*, vol. 288, pp. 43–47, 2016.
[69] A. Klimkowicz, K. Świerczek, A. Takasaki, J. Molenda, and B. Dabrowski, "Crystal structure and oxygen storage properties of BaLnMn2O5+ δ (Ln: Pr, Nd, Sm, Gd, Dy, Er and Y) oxides," *Materials Research Bulletin*, vol. 65, pp. 116–122, 2015.
[70] I. O. Troyanchuk, I. M. Kolesova, H. Szymczak, and A. Nabialek, "Preparation, magnetic and transport properties of A0. 66Ba0. 34MnO3- y (A= Pr, Nd, Sm, Eu, Gd) perovskites," *Journal of magnetism and magnetic materials*, vol. 176, no. 2–3, pp. 267–271, 1997.
[71] O. Yu. Gorbenko *et al.*, "Crystallographic, magnetic, and electrical properties of thin Re 0.6Ba0.4MnO3 epitaxial films (Re = La, Pr, Nd, Gd)," *Phys. Solid State*, vol. 46, no. 7, pp. 1255–1261, Jul. 2004, doi: 10.1134/1.1778450.
[72] T. Nakajima, H. Kageyama, H. Yoshizawa, K. Ohoyama, and Y. Ueda, "Ground State Properties of the *A* -site Ordered Manganites, *R* $BaMn_2O_6$ (*R* = La, Pr and Nd)," *J. Phys. Soc. Jpn.*, vol. 72, no. 12, pp. 3237–3242, Dec. 2003, doi: 10.1143/JPSJ.72.3237.
[73] Y. Ueda and T. Nakajima, "Novel structures and electromagnetic properties of the A-site-ordered/disordered manganites RBaMn2O6/R0. 5Ba0. 5MnO3 (R= Y and rare earth elements)," *Journal of Physics: Condensed Matter*, vol. 16, no. 11, p. S573, 2004.
[74] R. Mahesh, R. Mahendiran, A. K. Raychaudhuri, and C. N. R. Rao, "Effect of the internal pressure due to the A-site cations on the giant magnetoresistance and related properties of doped rare earth manganates, Ln1-xAxMnO3 (Ln= La, Nd, Gd, Y; A= Ca, Sr, Ba, Pb)," *Journal of Solid State Chemistry*, vol. 120, no. 1. Elsevier, pp. 204–207, 1995. Accessed: Dec. 09, 2024. [Online]. Available: https://www.sciencedirect.com/science/article/pii/S0022459685713989
[75] K. Das, T. Paramanik, and I. Das, "Large magnetocaloric effect in Ln0. 5Ca0. 5MnO3 (Ln= Gd, Dy) compounds: conseqence of magnetic precursor effect of rare earth ions," *Journal of Magnetism and Magnetic Materials*, vol. 374, pp. 707–710, 2015.
[76] K. V. Sarathy, S. Parashar, A. R. Raju, and C. N. R. Rao, "Hopping conduction in charge-ordered rare-earth manganates Ln1- xCaxMnO3 (Ln= rare earth)," *Solid state sciences*, vol. 4, no. 3, pp. 353–357, 2002.
[77] Y. Wang *et al.*, "High Temperature Thermoelectric Response of Electron-Doped $CaMnO_3$," *Chem. Mater.*, vol. 21, no. 19, pp. 4653–4660, Oct. 2009, doi: 10.1021/cm901766y.
[78] Y. Wang, Y. Sui, and W. Su, "High temperature thermoelectric characteristics of Ca0. 9R0. 1MnO3 (R= La, Pr,…, Yb)," *Journal of Applied Physics*, vol. 104, no. 9, 2008, Accessed: Dec. 09, 2024. [Online]. Available: https://pubs.aip.org/aip/jap/article/104/9/093703/388942
[79] Y. Wang, Y. Sui, J. Cheng, X. Wang, Z. Lu, and W. Su, "High Temperature Metal−Insulator Transition Induced by Rare-Earth Doping in Perovskite $CaMnO_3$," *J. Phys. Chem. C*, vol. 113, no. 28, pp. 12509–12516, Jul. 2009, doi: 10.1021/jp809049s.
[80] C. Moure and O. Peña, "Magnetic features in REMeO3 perovskites and their solid solutions (RE= rare-earth, Me= Mn, Cr)," *Journal of magnetism and magnetic materials*, vol. 337, pp. 1–22, 2013.
[81] J. Lan *et al.*, "High-Temperature Thermoelectric Behaviors of Fine-Grained Gd-Doped $CaMnO_3$ Ceramics," *Journal of the American Ceramic Society*, vol. 93, no. 8, pp. 2121–2124, Aug. 2010, doi: 10.1111/j.1551-2916.2010.03673.x.
[82] N. Haberkorn, S. Larregola, D. Franco, and G. Nieva, "Inhomogeneous ferrimagnetic-like behavior in Gd2/3Ca1/3MnO3 single crystals," *Journal of magnetism and magnetic materials*, vol. 321, no. 9, pp. 1133–1136, 2009.

[83] A. Beiranvand, J. Tikkanen, H. Huhtinen, and P. Paturi, “Electronic and magnetic phase diagram of polycrystalline Gd1- xCaxMnO3 manganites,” *Journal of Alloys and Compounds*, vol. 720, pp. 126–130, 2017.
[84] P. N. Santhosh, A. Arulraj, P. V. Vanitha, R. S. Singh, K. Sooryanarayana, and C. N. R. Rao, “Charge ordering in electron-doped manganates,” *Journal of Physics: Condensed Matter*, vol. 11, no. 5, p. L27, 1999.
[85] A. Srivastava and N. K. Gaur, “Bulk modulus and thermodynamic properties of electron-doped calcium manganate—Ca1- xRExMnO3,” *Journal of magnetism and magnetic materials*, vol. 321, no. 23, pp. 3854–3865, 2009.
[86] Y. Wang, Y. Sui, X. Wang, W. Su, and X. Liu, “Correlation of structural distortion with magnetic properties in electron-doped Ca0. 9R0. 1MnO3 perovskites (R= rare-earth),” *Journal of Applied Physics*, vol. 108, no. 6, 2010, Accessed: Dec. 09, 2024. [Online]. Available: https://pubs.aip.org/aip/jap/article/108/6/063928/348668
[87] L. Dhal, R. Nirmala, P. N. Santhosh, T. G. Kumary, and A. K. Nigam, “Bulk and nanocrystalline electron doped Gd0. 15Ca0. 85MnO3: Synthesis and magnetic characterization,” *Physica B: Condensed Matter*, vol. 448, pp. 300–303, 2014.
[88] R. R. Zhang, G. L. Kuang, X. Luo, and Y. P. Sun, “The effect of Gd-doping on the charge ordering state of Bi0. 3- xGdxCa0. 7MnO3 (0≤ x≤ 0.30),” *Journal of magnetism and magnetic materials*, vol. 321, no. 23, pp. 3933–3937, 2009.
[89] H. Taguchi, M. Nagao, and M. Shimada, “Metal-insulator transition in the system (Gd1-xCax) MnO2. 98,” *Journal of Solid State Chemistry*, vol. 82, no. 1, pp. 8–13, 1989.
[90] M. Rosić *et al.*, “Synthesis, structural and magnetic properties of nanostructured Ca0. 9Gd0. 1MnO3 obtained by modified glycine nitrate procedure (MGNP),” *Ceramics International*, vol. 37, no. 4, pp. 1313–1319, 2011.
[91] P. Negi, H. M. Agrawal, and R. C. Srivastava, “Structural, specific heat and magnetoresistive properties of Gd<SUB align="right">0.7Ca<SUB align="right">0.3MnO<SUB align="right">3,” *IJMATEI*, vol. 5, no. 3, p. 216, 2014, doi: 10.1504/IJMATEI.2014.064279.
[92] Y. Wang, Y. Sui, X. Wang, and W. Su, “Seebeck coefficient of Ln x Ca1−x MnO3 perovskites in paramagnetic state,” *Appl. Phys. A*, vol. 104, no. 1, pp. 135–142, Jul. 2011, doi: 10.1007/s00339-010-6081-6.
[93] V. V. Kharton, A. A. Yaremchenko, and E. N. Naumovich, “Research on the electrochemistry of oxygen ion conductors in the former Soviet Union. II. Perovskite-related oxides,” *Journal of Solid State Electrochemistry*, vol. 3, no. 6, pp. 303–326, Aug. 1999, doi: 10.1007/s100080050161.
[94] I. Medvedeva *et al.*, “Hydrostatic pressure effect on electrical and magnetic properties of electron-doped R0. 16Ca0. 84MnO3 (R= Pr, Gd, Eu),” *Physica B: Condensed Matter*, vol. 365, no. 1–4, pp. 114–120, 2005.
[95] L. Ling, J. Fan, L. Pi, Y. Ying, S. Tan, and Y. Zhang, “Ordering state and magnetism in highly doped manganite Gd0. 4Ca0. 6MnO3,” *Solid state communications*, vol. 144, no. 5–6, pp. 189–193, 2007.
[96] M. Rosic *et al.*, “INVESTIGATION OF THE STRUCTURE AND THE MAGNETIC BEHAVIOUR OF NANOSTRUCTURED Ca 1-xGdxMnO 3(x= 0. 05; 0. 1; 0. 15; 0. 2) OBTAINED BY MODIFIED GLYCINE NITRATE PROCEDURE,” *Ceramics International*, vol. 39, no. 2, pp. 1853–1861, 2013.
[97] M. Pękała and V. A. Drozd, “Magneto-transport study of manganites (La0. 75- xGdx) Ca0. 25MnO3,” *Journal of alloys and compounds*, vol. 437, no. 1–2, pp. 12–15, 2007.

[98] R. Sinclair *et al.*, “Canted magnetic ground state of quarter-doped manganites R0. 75Ca0. 25MnO3 (R= Y, Tb, Dy, Ho, and Er),” *Journal of Physics: Condensed Matter*, vol. 29, no. 6, p. 065802, 2016.
[99] N. H. Linh, N. T. Trang, N. T. Cuong, P. H. Thao, and B. T. Cong, “Influence of doped rare earth elements on electronic properties of the R0. 25Ca0. 75MnO3 systems,” *Computational materials science*, vol. 50, no. 1, pp. 2–5, 2010.
[100] R. Choithrani and N. K. Gaur, “Analysis of low temperature specific heat in Nd0. 5Sr0. 5MnO3 and R0. 5Ca0. 5MnO3 (R= Nd, Sm, Dy and Ho) compounds,” *Journal of magnetism and magnetic materials*, vol. 320, no. 24, pp. 3384–3389, 2008.
[101] A. Martinelli, M. Ferretti, C. Castellano, M. R. Cimberle, R. Masini, and C. Ritter, “Neutron powder diffraction investigation on the crystal and magnetic structure of (Ho0.50+xCa0.50−x)(Mn1−xCrx)O3,” *J. Phys.: Condens. Matter*, vol. 23, no. 41, p. 416005, Sep. 2011, doi: 10.1088/0953-8984/23/41/416005.
[102] J. López and O. F. De Lima, “Specific heat at high temperature and magnetic measurements in Nd0. 5Sr0. 5MnO3 and R0. 5Ca0. 5MnO3 (R= Nd, Sm, Dy and Ho) samples,” *Journal of alloys and compounds*, vol. 369, no. 1–2, pp. 227–230, 2004.
[103] D. Sousa, M. R. Nunes, C. Silveira, I. Matos, A. B. Lopes, and M. M. Jorge, “Ca-site substitution induced a metal–insulator transition in manganite CaMnO3,” *Materials Chemistry and Physics*, vol. 109, no. 2–3, pp. 311–319, 2008.
[104] P. H. Isasi, M. E. Lopes, M. R. Nunes, and M. M. Jorge, “Low-temperature synthesis of nanocrystalline Ca1- xHoxMnO3- δ (0⩽ x⩽ 0.3) powders,” *Journal of Physics and Chemistry of Solids*, vol. 70, no. 2, pp. 405–411, 2009.
[105] Y. Wang *et al.*, “High Temperature Thermoelectric Response of Electron-Doped $CaMnO_3$,” *Chem. Mater.*, vol. 21, no. 19, pp. 4653–4660, Oct. 2009, doi: 10.1021/cm901766y.
[106] Y. Wang, Y. Sui, and W. Su, “High temperature thermoelectric characteristics of Ca0. 9R0. 1MnO3 (R= La, Pr,…, Yb),” *Journal of Applied Physics*, vol. 104, no. 9, 2008, Accessed: Dec. 09, 2024. [Online]. Available: https://pubs.aip.org/aip/jap/article/104/9/093703/388942
[107] Y. Wang, Y. Sui, X. Wang, W. Su, and X. Liu, “Correlation of structural distortion with magnetic properties in electron-doped Ca0. 9R0. 1MnO3 perovskites (R= rare-earth),” *Journal of Applied Physics*, vol. 108, no. 6, 2010, Accessed: Dec. 09, 2024. [Online]. Available: https://pubs.aip.org/aip/jap/article/108/6/063928/348668
[108] A. Martinelli and M. Ferretti, “The crystal structure of (Ho0. 50Ca0. 50) MnO3 and its evolution with Cr doping: A Rietveld refinement investigation,” *Powder diffraction*, vol. 20, no. 1, pp. 22–26, 2005.
[109] T. Kobayashi *et al.*, “Metal-insulator transition and thermoelectric properties in the system (R1- xCax) MnO3- δ (R: Tb, Ho, Y),” *Journal of solid state chemistry*, vol. 92, no. 1, pp. 116–129, 1991.
[110] D. Flahaut *et al.*, “Thermoelectrical properties of A-site substituted Ca1- xRexMnO3 system,” *Journal of Applied Physics*, vol. 100, no. 8, 2006, Accessed: Dec. 09, 2024. [Online]. Available: https://pubs.aip.org/aip/jap/article/100/8/084911/931019
[111] K. Yoshii, H. Abe, and N. Ikeda, “Structure, magnetism and transport of the perovskite manganites Ln0. 5Ca0. 5MnO3 (Ln= Ho, Er, Tm, Yb and Lu),” *Journal of Solid State Chemistry*, vol. 178, no. 12, pp. 3615–3623, 2005.
[112] T. Zeiske, K. Hagdorn, D. Hohlwein, J. Ihringer, W. Prandl, and H. Ritter, “Structure and magnetism of Ho0. 2Ca0. 8MnO3,” *Physica B: Condensed Matter*, vol. 276, pp. 624–625, 2000.

[113] J. López, O. F. De Lima, P. N. Lisboa-Filho, and F. M. Araujo-Moreira, "Specific heat at low temperatures and magnetic measurements in Nd 0.5 Sr 0.5 MnO 3 and R 0.5 Ca 0.5 MnO 3 (R = Nd , Sm, Dy, and Ho) samples," *Phys. Rev. B*, vol. 66, no. 21, p. 214402, Dec. 2002, doi: 10.1103/PhysRevB.66.214402.
[114] Y. Wang, Y. Sui, X. Wang, and W. Su, "Seebeck coefficient of Ln x Ca1−x MnO3 perovskites in paramagnetic state," *Appl. Phys. A*, vol. 104, no. 1, pp. 135–142, Jul. 2011, doi: 10.1007/s00339-010-6081-6.
[115] Y. Wang, Y. Sui, J. Cheng, X. Wang, Z. Lu, and W. Su, "High Temperature Metal−Insulator Transition Induced by Rare-Earth Doping in Perovskite $CaMnO_3$," *J. Phys. Chem. C*, vol. 113, no. 28, pp. 12509–12516, Jul. 2009, doi: 10.1021/jp809049s.
[116] R. Vengadesh Kumara Mangalam and A. Sundaresan, "Structural, magnetic and magnetotransport properties of La0.7−x Ce x Ba0.3MnO3 (x = 0.0–0.4)," *J Chem Sci*, vol. 118, no. 1, pp. 99–103, Jan. 2006, doi: 10.1007/BF02708771.
[117] S. Bošković, J. Dukić, B. Matović, L. Živković, M. Vlajić, and V. Krstić, "Nanopowders properties and sintering of CaMnO3 solid solutions," *Journal of alloys and compounds*, vol. 463, no. 1–2, pp. 282–287, 2008.
[118] R. Zalecki *et al.*, "Photoemission band structure of La0. 7 (Ca, Ce) 0.3 MnO3 thin films and a La0. 9Ca0. 1CoO3 crystal," *Journal of alloys and compounds*, vol. 442, no. 1–2, pp. 296–298, 2007.
[119] S.-J. Sun, C.-H. Lin, P.-Y. Liao, and H. Chou, "Magnetic coupling in the mixture of different doped colossal magnetoresistances," *Applied physics letters*, vol. 89, no. 7, 2006, Accessed: Dec. 10, 2024. [Online]. Available: https://pubs.aip.org/aip/apl/article/89/7/072502/332332
[120] J. E. Gayone, M. Abbate, G. Alejandro, D. G. Lamas, M. Tovar, and G. Zampieri, "Ce valence in La0. 47Ce0. 20Ca0. 33MnO3," *Journal of alloys and compounds*, vol. 369, no. 1–2, pp. 252–255, 2004.
[121] S. Y. Wu *et al.*, "Crystal structure and magnetic ordering of Mn and Ce in La0. 7Ce0. 15Ca0. 15MnO3," *Journal of Physics: Condensed Matter*, vol. 14, no. 47, p. 12585, 2002.
[122] V. Spasojevic *et al.*, "Magnetic properties of nanosized mixed valent manganites CaMnO3 and Ca0. 7La0. 3Mn1- xCexO3 (x= 0; 0.2)," *Journal of alloys and compounds*, vol. 442, no. 1–2, pp. 197–199, 2007.
[123] D. Varshney, I. Mansuri, N. Kaurav, W. Q. Lung, and Y. K. Kuo, "Influence of Ce doping on electrical and thermal properties of La {sub 0.7-x} Ce {sub x} Ca {sub 0.3} MnO {sub 3}(0.0 {<=} x {<=} 0.7) manganites," *Journal of Magnetism and Magnetic Materials*, vol. 324, 2012, Accessed: Dec. 10, 2024. [Online]. Available: https://www.osti.gov/etdeweb/biblio/21610999
[124] N. N. Loshkareva and E. V. Mostovshchikova, "Electron-doped manganites based on CaMnO3," *Phys. Metals Metallogr.*, vol. 113, no. 1, pp. 19–38, Jan. 2012, doi: 10.1134/S0031918X12010073.
[125] T. A. Ho *et al.*, "Magnetic and magnetocaloric properties of La0. 6Ca0. 4- xCexMnO3," *Journal of Magnetism and Magnetic Materials*, vol. 438, pp. 52–59, 2017.
[126] R. Chihoub, A. Amira, N. Mahamdioua, S. P. Altintas, A. Varilci, and C. Terzioglu, "Magnetoresistive properties of cerium doped La0. 7Ca0. 3MnO3 manganites," *Physica B: Condensed Matter*, vol. 492, pp. 11–15, 2016.
[127] G. Alejandro, L. B. Steren, A. Caneiro, J. Cartes, E. E. Vogel, and P. Vargas, "Barkhausen-like steps and magnetic frustration in doped La 0.67 − x A x Ca 0.33 Mn O 3 (A = Ce , Y)," *Phys. Rev. B*, vol. 73, no. 5, p. 054427, Feb. 2006, doi: 10.1103/PhysRevB.73.054427.

[128] A. Khare, A. Bodhaye, D. Bhargava, R. J. Choudhary, and S. P. Sanyal, “Study of structural, transport and magneto-resistive properties of La0. 7Ca0. 3- xCexMnO3 (0≤ x≤ 0.2),” *Physica B: Condensed Matter*, vol. 404, no. 20, pp. 3602–3607, 2009.
[129] Y. Li *et al.*, “Giant magnetoresistance in bulk La {sub 0.6} Mg {sub 0.4} MnO {sub 3},” *Journal of Materials Research*, vol. 12, no. 10, 1997, Accessed: Jun. 03, 2024. [Online]. Available: https://www.osti.gov/biblio/554347
[130] R. Selmi, W. Cherif, A. R. Sarabando, N. M. Ferreira, and L. Ktari, “Enhanced relative cooling power of lanthanum-deficiency manganites La0.77−xMg0.23MnO3 (0 ≤ x ≤ 0.2): structural, magnetic and magnetocaloric properties,” *J Mater Sci: Mater Electron*, vol. 33, no. 3, pp. 1703–1723, Jan. 2022, doi: 10.1007/s10854-022-07726-8.
[131] J. H. Zhao, H. P. Kunkel, X. Z. Zhou, and G. Williams, “Magnetic and transport behavior of electron-doped La 1 − x Mg x MnO 3 (0.45 <~ x <~ 0 . 6),” *Phys. Rev. B*, vol. 66, no. 18, p. 184428, Nov. 2002, doi: 10.1103/PhysRevB.66.184428.
[132] C. Zhang *et al.*, “The effect of A-site substitution by Sr, Mg and Ce on the catalytic performance of LaMnO3 catalysts for the oxidation of vinyl chloride emission,” *Applied Catalysis B: Environmental*, vol. 134, pp. 310–315, 2013.
[133] B. K. Kasenov *et al.*, “La2M 3 II Mn4O12 (M = Mg, Ca, Sr, or Ba) manganites: Synthesis and X-ray diffraction study,” *Russ. J. Inorg. Chem.*, vol. 52, no. 10, pp. 1514–1515, Oct. 2007, doi: 10.1134/S0036023607100063.
[134] D. Dilner, D. Pavlyuchkov, T. Zienert, L. Kjellqvist, and O. Fabrichnaya, “Thermodynamics of the Mg-Mn-O system—modeling and heat capacity measurements,” *J Am Ceram Soc*, vol. 100, no. 4, pp. 1661–1672, Apr. 2017, doi: 10.1111/jace.14686.
[135] J. H. Zhao, H. P. Kunkel, X. Z. Zhou, and G. Williams, “Magnetic and transport properties, and the phase diagram of hole-doped La1- xMgxMnO3 (x≤ 0.4),” *Journal of Physics: Condensed Matter*, vol. 13, no. 41, p. 9349, 2001.
[136] G. I. Supelano, A. J. Barón-González, A. S. Santos, C. Ortíz, J. A. M. Gómez, and C. A. P. Vargas, “Effect of Mg addition on LaMnO3 ceramic system,” *Journal of materials research and technology*, vol. 7, no. 1, pp. 77–81, 2018.
[137] X. Zhu *et al.*, “La0. 8M0. 2MnO3 (M= Ba, Ca, Ce, Mg and Sr) perovskite catalysts for plasma-catalytic oxidation of ethyl acetate,” *Catalysis Communications*, vol. 92, pp. 35–39, 2017.
[138] X. Z. Zhou, H. P. Kunkel, J. H. Zhao, P. A. Stampe, and G. Williams, “Evidence for an enhanced magnetoresistance accompanying a continuous phase transition in semiconducting La 0.67 Mg 0.33 MnO 3,” *Phys. Rev. B*, vol. 56, no. 20, pp. R12714–R12717, Nov. 1997, doi: 10.1103/PhysRevB.56.R12714.
[139] S. M. Ramay, A. Mahmood, S. Atiq, and A. N. AlHazaa, “Study of divalent elements (Mg, Sr and Ba)-doped $LaMnO_3$ nano-manganites,” *Int. J. Mod. Phys. B*, vol. 30, no. 06, p. 1650020, Mar. 2016, doi: 10.1142/S021797921650020X.
[140] L. Liu, Z. C. Xia, and S. L. Yuan, “Effect of Mg doping on the transport properties and magnetoresistance of La2/3Ca1/3MnO3 prepared in low temperature,” *Materials Science and Engineering: B*, vol. 128, no. 1–3, pp. 50–52, 2006.
[141] T. Fang, E. Lu, and H. Ho, “Comparison of the Magnetic, Conduction, and Magnetotransport Behaviors of Ba^{2+} - and Al^{3+} -Doped $La_{0.67}$ $Ca_{0.33}$ MnO_3,” *Journal of the American Ceramic Society*, vol. 88, no. 12, pp. 3412–3418, Dec. 2005, doi: 10.1111/j.1551-2916.2005.00610.x.
[142] H. K. Singh, A. K. Gupta, P. K. Siwach, and O. N. Srivastava, “Effect of Ba doping on structure and magneto-transport properties of layered manganite La1. 4Ca1. 6- xBaxMn2O7,” *Journal of magnetism and magnetic materials*, vol. 292, pp. 483–489, 2005.

[143] E. A.Gan’shina, “Electronic structure and magnetooptical properties of an La0.7Ca0.25Ba0.05MnO3 single crystal | Crystallography Reports.” Accessed: Dec. 10, 2024. [Online]. Available: https://link.springer.com/article/10.1134/1.1578135
[144] Y. Jiang and S. L. Yuan, “STRUCTURAL AND TRANSPORT PROPERTIES OF $La_{0.5}$ $Ca_{0.5-x}$ Ba_x MnO_3 ($0 \leq x \leq 0.5$),” *Mod. Phys. Lett. B*, vol. 15, no. 21, pp. 935–940, Sep. 2001, doi: 10.1142/S0217984901002701.
[145] V. N. Smolyaninova *et al.*, “Effect of A-site cation disorder on charge ordering and ferromagnetism of La0. 5Ca0. 5- yBayMnO3,” *Journal of magnetism and magnetic materials*, vol. 248, no. 2, pp. 348–354, 2002.
[146] L. M. Rodríguez-Martínez, H. Ehrenberg, and J. P. Attfield, “Cation size variance effects in high-tolerance factor Ln0. 7M0. 3MnO3 perovskites,” *Journal of Solid State Chemistry*, vol. 148, no. 1, pp. 20–25, 1999.
[147] F. Ayadi *et al.*, “Preparation of nanostructured La0. 7Ca0. 3- xBaxMnO3 ceramics by a combined sol–gel and spark plasma sintering route and resulting magnetocaloric properties,” *Journal of Magnetism and Magnetic Materials*, vol. 381, pp. 215–219, 2015.
[148] V. B. Naik, M. C. Lam, and R. Mahendiran, “Detection of structural and magnetic transitions in La0. 67Ba0. 23Ca0. 1MnO3 using the rf resonance technique,” *Journal of magnetism and magnetic materials*, vol. 322, no. 18, pp. 2754–2757, 2010.
[149] I. Dhiman, A. Das, and A. K. Nigam, “Structural and magnetic ordering in La0. 5Ca0. 5- xBaxMnO3 (0< x≤ 0.5) manganite,” *Journal of Physics: Condensed Matter*, vol. 21, no. 38, p. 386002, 2009.
[150] P. Mandal, P. Choudhury, and B. Ghosh, “Electronic transport in ferroelectric-ferromagnetic composites La 5 ⁄ 8 (Ba , Ca) 3 ⁄ 8 MnO 3 : LuMnO 3,” *Phys. Rev. B*, vol. 74, no. 9, p. 094421, Sep. 2006, doi: 10.1103/PhysRevB.74.094421.
[151] A. M. Gomes, M. S. Reis, A. P. Guimaraes, P. B. Tavares, J. P. Araujo, and V. S. Amaral, “Magnetocaloric effect on the Pr0. 43Gd0. 25Ca0. 32MnO3 manganite,” *Journal of magnetism and magnetic materials*, vol. 272, pp. 2385–2386, 2004.
[152] K. R. Mavani and P. L. Paulose, “A comparative study on metamagnetic transitions in half-doped Pr0. 45La0. 05Ca0. 5MnO3 and Pr0. 45Gd0. 05Ca0. 5MnO3 manganites,” *Physica B: Condensed Matter*, vol. 378, pp. 504–506, 2006.
[153] R. Ganguly, A. Maignan, M. Hervieu, C. Martin, and B. Raveau, “Phase stability and properties of the hole doped perovskite cobaltites (La1- xRx) 0.7 Ba0. 3CoO3- δ (0.0≤ x≤ 1.0; R= Pr, Nd),” *Solid state communications*, vol. 121, no. 9–10, pp. 537–542, 2002.
[154] G. L. Reddy, Y. K. Lakshmi, S. M. Rao, and P. V. Reddy, “Thermopower studies of rare earth doped lanthanum barium manganites,” *Journal of magnetism and magnetic materials*, vol. 362, pp. 20–26, 2014.
[155] R. Cherif, E. K. Hlil, M. Ellouze, F. Elhalouani, and S. Obbade, “Study of magnetic and magnetocaloric properties of La0.6Pr0.1Ba0.3MnO3 and La0.6Pr0.1Ba0.3Mn0.9Fe0.1O3 perovskite-type manganese oxides,” *J Mater Sci*, vol. 49, no. 24, pp. 8244–8251, Dec. 2014, doi: 10.1007/s10853-014-8533-4.
[156] J. Khelifi, A. Tozri, E. Dhahri, and E. K. Hlil, “Influence of Pr-doped manganite on critical behavior of La0. 7- xPrxBa0. 3MnO3 (x= 0.00, 0.1, 0.2),” *Journal of magnetism and magnetic materials*, vol. 349, pp. 149–155, 2014.
[157] A. K. Kundu, M. M. Seikh, K. Ramesha, and C. N. R. Rao, “Novel effects of size disorder on the electronic and magnetic properties of rare earth manganates of the type La0. 7- xLnxBa0.

3MnO3 (Ln= Pr, Nd, Gd or Dy) with large average radius of the A-site cations," *Journal of Physics: Condensed Matter*, vol. 17, no. 26, p. 4171, 2005.

[158] S. Zouari, M. L. Kahn, M. Ellouze, and F. Elhalouani, "Effect of iron substitution on the physico-chemical properties of Pr0.6La0.1Ba0.3Mn1−xFexO3 manganites (with 0 ≤ x ≤0.3," *Eur. Phys. J. Plus*, vol. 130, no. 8, p. 177, Aug. 2015, doi: 10.1140/epjp/i2015-15177-2.

[159] M. Oumezzine, H. B. Sales, A. Selmi, and E. K. Hlil, "Pr 3+ doping at the A-site of La 0.67 Ba 0.33 MnO 3 nanocrystalline material: assessment of the relationship between structural and physical properties and Bean–Rodbell model simulation of disorder effects," *RSC advances*, vol. 9, no. 44, pp. 25627–25637, 2019.

[160] E. Oumezzine, S. Hcini, E.-K. Hlil, E. Dhahri, and M. Oumezzine, "Effect of Ni-doping on structural, magnetic and magnetocaloric properties of La0. 6Pr0. 1Ba0. 3Mn1- xNixO3 nanocrystalline manganites synthesized by Pechini sol–gel method," *Journal of alloys and compounds*, vol. 615, pp. 553–560, 2014.

[161] R. Hamdi, J. Khelifi, I. Walha, E. Dhahri, and E. K. Hlil, "Impact of Titanium Doping on Structural, Magnetic, and Magnetocaloric Properties and Order of Transition in La0.5Pr0.3Ba0.2Mn1-xTixO3 (x = 0.0 and 0.1) Manganite," *J Supercond Nov Magn*, vol. 32, no. 11, pp. 3679–3690, Nov. 2019, doi: 10.1007/s10948-019-5142-0.

[162] G. L. Reddy, Y. K. Lakshmi, S. M. Rao, and P. V. Reddy, "Thermopower studies of rare earth doped lanthanum barium manganites," *Journal of magnetism and magnetic materials*, vol. 362, pp. 20–26, 2014.

[163] A. K. Kundu, M. M. Seikh, K. Ramesha, and C. N. R. Rao, "Novel effects of size disorder on the electronic and magnetic properties of rare earth manganates of the type La0. 7- xLnxBa0. 3MnO3 (Ln= Pr, Nd, Gd or Dy) with large average radius of the A-site cations," *Journal of Physics: Condensed Matter*, vol. 17, no. 26, p. 4171, 2005.

[164] A. K. Kundu, K. Ramesha, R. Seshadri, and C. N. R. Rao, "Magnetic and electron transport properties of the rare earth cobaltates, La0. 7- xLnxCa0. 3CoO3 (Ln= Pr, Nd, Gd and Dy): a case of phase separation," *Journal of Physics: Condensed Matter*, vol. 16, no. 45, p. 7955, 2004.

[165] J. R. Sun, G. H. Rao, and J. K. Liang, "Crystal structure and electronic transport property of perovskite manganese oxides with a fixed tolerance factor," *Applied physics letters*, vol. 70, no. 14, pp. 1900–1902, 1997.

[166] T. Terai, T. Kakeshita, T. Fukuda, K. Kindo, M. Honda, and K. Kishio, "Electronic and magnetic properties and phase diagrams of (La$_{1-X}$ R$_X$)$_{0.7}$ Ca$_{0.3}$ MnO$_3$ compounds with R = Sm, Gd, Ho or Er," *Philosophical Magazine B*, vol. 82, no. 10, pp. 1099–1111, Jul. 2002, doi: 10.1080/13642810208223152.

[167] L. Sudheendra and C. N. R. Rao, "Electronic phase separation in the rare-earth manganates (La1- xLnx) 0.7 Ca0. 3MnO3 (Ln= Nd, Gd and Y)," *Journal of Physics: Condensed Matter*, vol. 15, no. 19, p. 3029, 2003.

[168] A. Aslam, S. K. Hasanain, M. J. Akhtar, and M. Nadeem, "Stabilization of the ferromagnetic metallic state in rare earth-doped La0. 49X0. 01Ca0. 50MnO3+ δ (X= Nd, Sm, Gd and Yb)," *Journal of magnetism and magnetic materials*, vol. 301, no. 1, pp. 79–87, 2006.

[169] M. Pękała and V. A. Drozd, "Magneto-transport study of manganites (La0. 75- xGdx) Ca0. 25MnO3," *Journal of alloys and compounds*, vol. 437, no. 1–2, pp. 12–15, 2007.

[170] S. P. Altintas, A. Amira, A. Varilci, and C. Terzioglu, "Influence of Gd-doping in La0. 7Ca0. 3MnO3 on its structural and magneto-electrical properties," *Journal of magnetism and magnetic materials*, vol. 324, no. 7, pp. 1331–1336, 2012.

[171] A. Krichene, W. Boujelben, and A. Cheikhrouhou, “Structural, magnetic and magnetocaloric properties in La0. 5- xRexCa0. 5MnO3 manganites (x= 0; 0.1 and Re= Gd, Eu and Dy),” *Journal of alloys and compounds*, vol. 550, pp. 75–82, 2013.
[172] R. L. Zhang *et al.*, “The effect of Ho-doping and Ho-adding on electronic transport and magnetic properties of La0. 67Ca0. 33MnO3,” *Journal of magnetism and magnetic materials*, vol. 281, no. 2–3, pp. 318–325, 2004.
[173] T. Terai, T. Kakeshita, T. Fukuda, K. Kindo, M. Honda, and K. Kishio, “Electronic and magnetic properties and phase diagrams of $(La_{1-X} R_X)_{0.7} Ca_{0.3} MnO_3$ compounds with R = Sm, Gd, Ho or Er,” *Philosophical Magazine B*, vol. 82, no. 10, pp. 1099–1111, Jul. 2002, doi: 10.1080/13642810208223152.
[174] R. D. Shannon, “Revised effective ionic radii and systematic studies of interatomic distances in halides and chalcogenides,” *Foundations of Crystallography*, vol. 32, no. 5, pp. 751–767, 1976.
